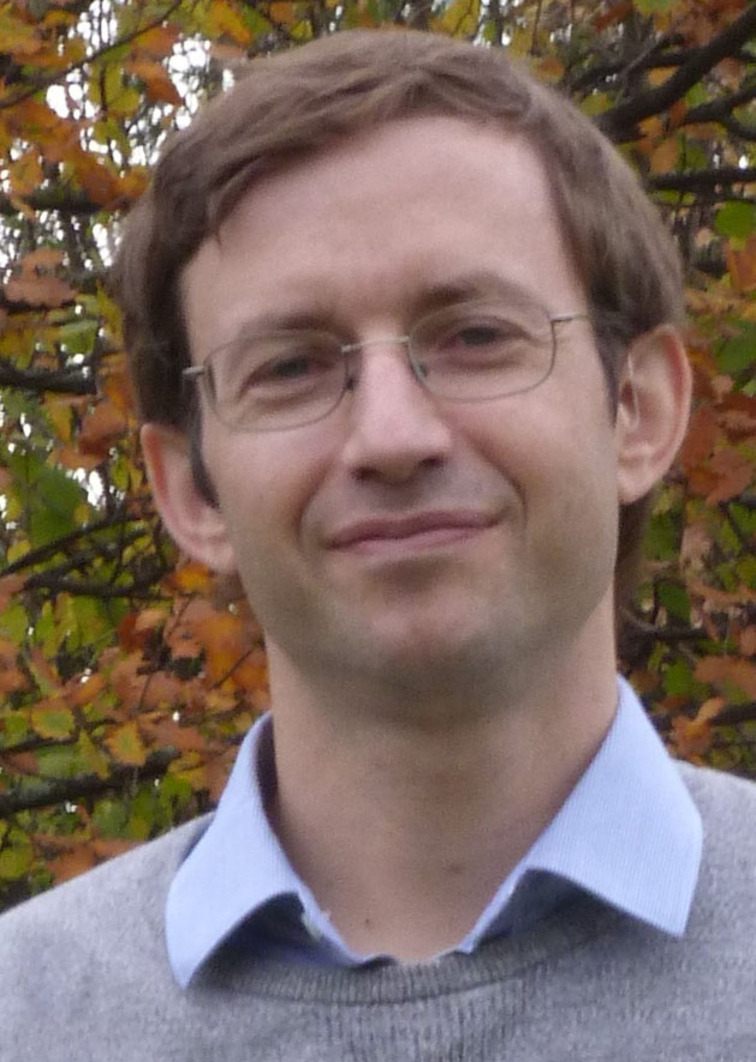# Hypervalent Iodine‐Mediated Late‐Stage Peptide and Protein Functionalization

**DOI:** 10.1002/anie.202112287

**Published:** 2021-12-08

**Authors:** Emmanuelle M. D. Allouche, Elija Grinhagena, Jerome Waser

**Affiliations:** ^1^ Laboratory of Catalysis and Organic Synthesis Institute of Chemical Sciences and Engineering Ecole Polytechnique Fédérale de Lausanne, EPFL, SB ISIC, LCSO, BCH 1402 1015 Lausanne Switzerland

**Keywords:** bioconjugation, hypervalent iodine, late-stage functionalization, peptides, proteins

## Abstract

Hypervalent iodine compounds are powerful reagents for the development of novel transformations. As they exhibit low toxicity, high functional group tolerance, and stability in biocompatible media, they have been used for the functionalization of biomolecules. Herein, we report recent advances up to June 2021 in peptide and protein modification using hypervalent iodine reagents. Their use as group transfer or oxidizing reagents is discussed in this Minireview, including methods targeting polar, aromatic, or aliphatic amino acids and peptide termini.

## Graphical abstract

Hypervalent iodine reagents are commonly used for the development of novel transformations. As they are highly reactive and biocompatible, they have been employed more recently for the functionalization of biomolecules. This Minireview discusses the development of hypervalent iodine reagents for the modification of peptides and proteins as well as their application, such as for labeling and peptide stapling.

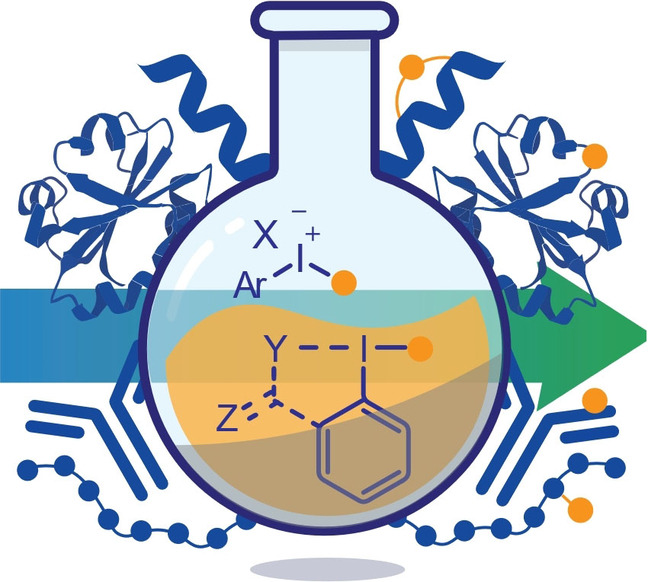

## 1 Introduction

Peptides and proteins are increasingly considered as drug candidates by established pharmaceutical companies.^[1]^ Around 80 peptide therapeutics are currently on the market, more than 150 peptides in clinical development, and 400–600 in preclinical studies.^[1i]^ Therefore, the need for novel bioconjugation strategies is constantly in demand to improve the properties of biomolecules or to study their biological function.^[2]^ However, the number of chemical transformations suitable for effective peptide and protein functionalization is still limited. They represent unique challenges for synthetic chemists, because of the range of functional groups present. In addition, these transformations need to be selective at a single site, proceed with fast reaction rates, operate under biologically compatible conditions, and should provide stable bioconjugates with near complete conversion.^[3]^

**Figure 1:**
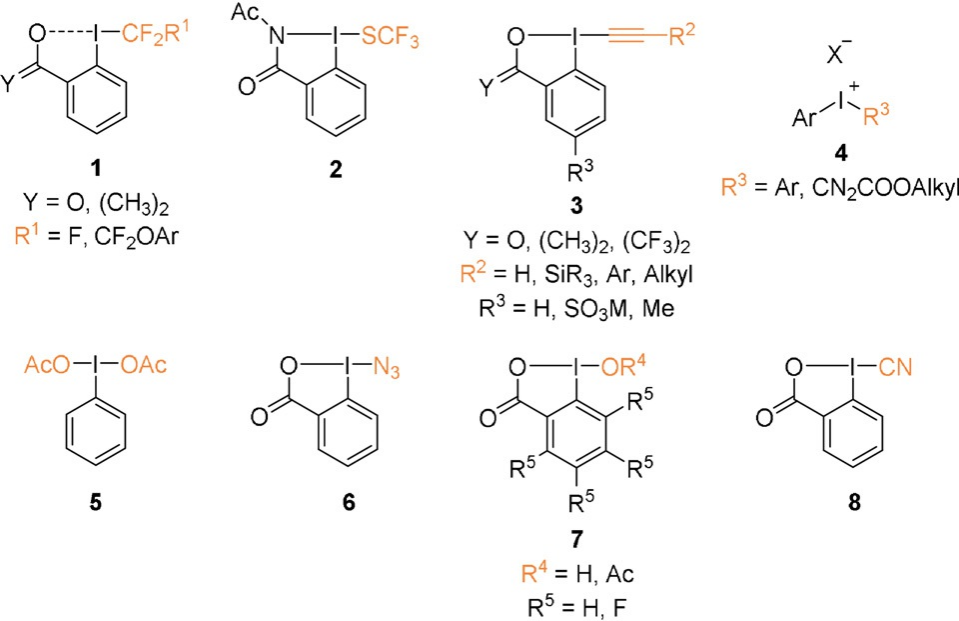
General representation of hypervalent iodine reagents used for peptide and protein functionalization.

Hypervalent iodine reagents have recently emerged as powerful tools for the functionalization of peptides and proteins. These reactive compounds have high functional group tolerance, are stable in biocompatible media,^[4]^ and are relatively nontoxic. Over the years, a variety of acyclic and cyclic hypervalent iodine reagents have been developed. Many of them are known for their oxidizing character, while others have been used as electrophilic group transfer reagents. Therefore, they can react with nucleophilic and oxidizable amino acids and can be used for the development of new methods for biomolecule functionalization (Figure 1).^[5]^

This Minireview depicts the recent advances up to June 2021 in the field of hypervalent iodine‐mediated modification of peptides and proteins. The functionalization of single amino acids and hypervalent iodine‐mediated peptide syntheses or cleavages will not be discussed. This Minireview is divided into four parts based on the targeted residue: sulfur‐containing, aromatic, or aliphatic amino acids or peptide termini.

## 2 Sulfur‐Containing Amino Acids

### 2.1 Cysteine

Cysteine (Cys) is one of the most targeted residues for the modification of biomolecules.[^6^, ^7^] Its low natural abundance and high nucleophilicity enable fast and efficient site‐selective bioconjugation methods, particularly in basic media.^[7]^

Hypervalent reagents **1**, **2**, and **3** have been used by the Togni and Zhang groups as well as our group, respectively, for the fluoroalkylation, thiolation, and alkynylation of thiols.[^8^, ^9^, ^10^] The methods have been then extended to peptides and proteins.

#### 2.1.1 Fluoroalkylation of Cys

**Scheme 1:**
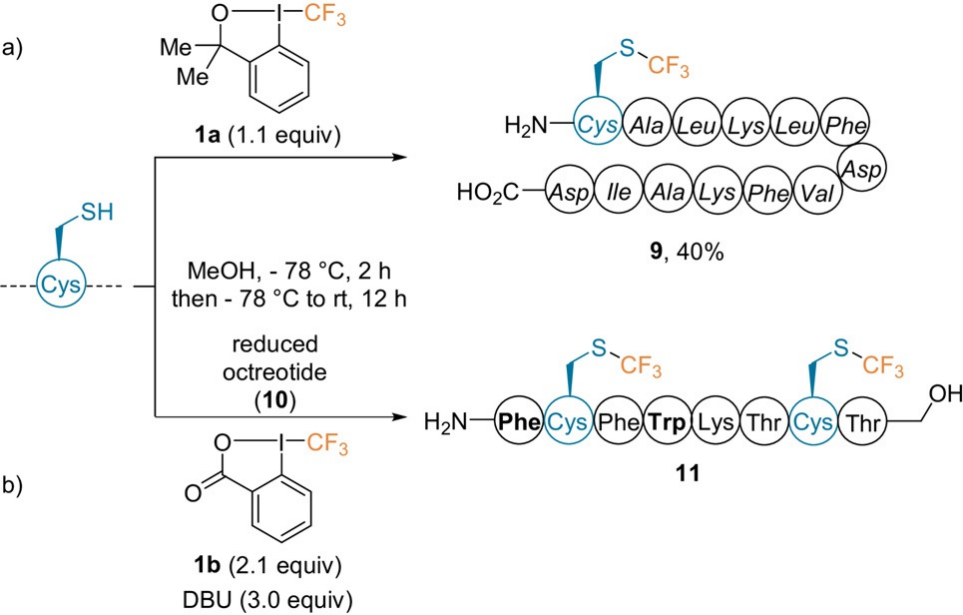
Selected examples of the trifluoromethylation of Cys‐containing peptides. Amino acids in italic are β‐amino acids. Amino acids in bold are d‐amino acids.

The introduction of fluorinated groups onto biomolecules in a mild and selective manner has attracted growing interest, as an improved in vivo stability can be achieved.^[11]^ To perform trifluoromethylation reactions, the Togni group developed cyclic hypervalent iodine reagents: the trifluoromethyl benziodoxol(on)es **1 a** and **1 b** (Scheme 1).[^5d^, ^8^, ^12^] In a collaboration with the Seebach group, Cys residues in peptides (up to 13‐residues long) containing α‐ and β‐amino acids were trifluoromethylated in good yields using **1 a** (**9**, Scheme 1 a).^[13]^ When applicable, it was necessary to reduce the disulfide bridge before the addition of the hypervalent iodine reagent, as it was inert to the reaction conditions. This transformation was also applied to the reduced (ring‐opened) peptide‐based drug octreotide **10** (Scheme 1 b).^[13]^ Reagent **1 b** was used along with 1,8‐diazabicyclo[5.4.0]undec‐7‐ene (DBU) to yield compound **11** as the only observed product. In the absence of DBU, trifluoromethylation of the tryptophan side chains was observed, while unprotected nucleophilic residues, including free amino and carboxylic groups, stayed intact.

**Scheme 2:**
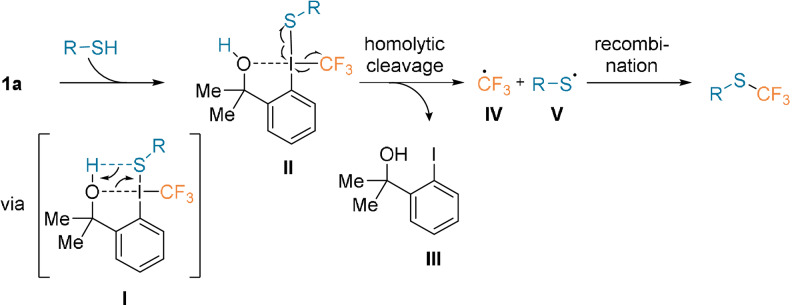
Most plausible mechanism for the trifluoromethylation of thiols.

The mechanism was investigated by experimental and computational studies using reagent **1 a** and thiophenol under standard reaction conditions (without additive; Scheme 2).^[14]^ A radical pathway is the most plausible, as the CF_3_ and thiyl radical species were intercepted by scavenging and trapping experiments. The disulfide and CF_3_H by‐products were also observed. Protonation of **1 a** is essential for the reaction to proceed. In the most plausible mechanism, the protonation would occur simultaneously with I−S bond formation (intermediate **I**), thereby yielding **II**. A Hammett plot, which shows the formation of a small negative charge on the sulfur atom during the rate‐determining step, supports this pathway. After homolytic cleavage, generation, and recombination of CF_3_ (**IV**) and thiyl (**V**) radicals, the desired compound would be generated. Other possible mechanisms start by the protonation of **1 a** by an external proton source. The *S*‐trifluoromethylated compound would then be formed after homolytic cleavage, recombination, and deprotonation.

**Scheme 3:**
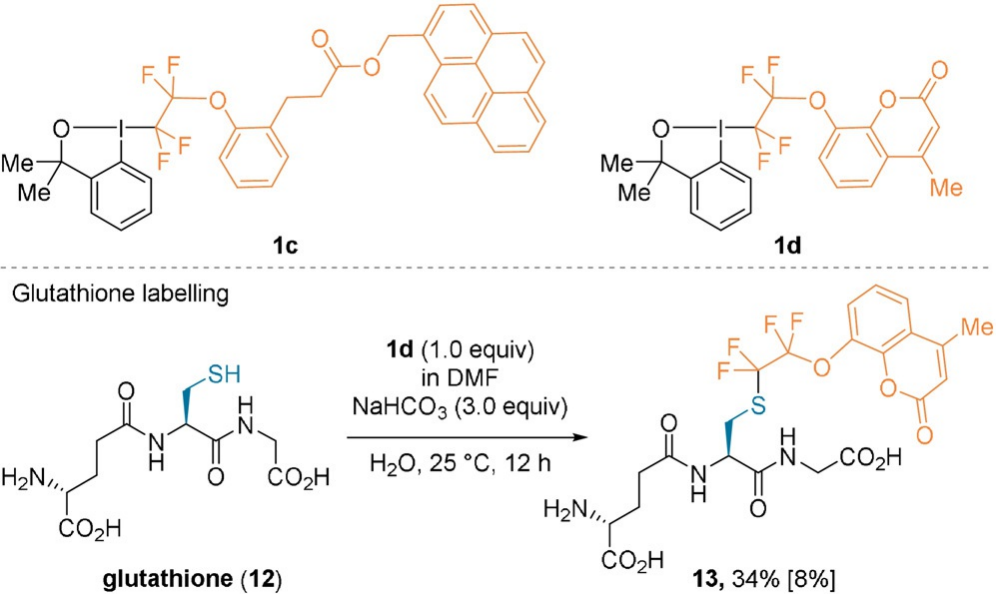
Selected examples of perfluoroethyl‐substituted reagents and application to glutathione labeling. The yield of the isolated product as a trifluoroacetate salt is given in brackets.

The Togni and Beier groups later developed a series of hypervalent iodine reagents bearing functionalized perfluoroethyl groups (Scheme 3).^[15]^ These compounds present a reactivity similar to their trifluoromethylated analogues and react with Cys residues. A wide range of reagents were synthesized, among them **1 c** and **1 d** bearing pyrene‐ or coumarin‐based chromophores.[^15^, ^16^] Glutathione (**12**) was labeled in moderate yields (34 % by ^19^F NMR spectroscopy and 8 % of the isolated product) using reagent **1 d** in an aqueous basic media at room temperature.

**Scheme 4:**
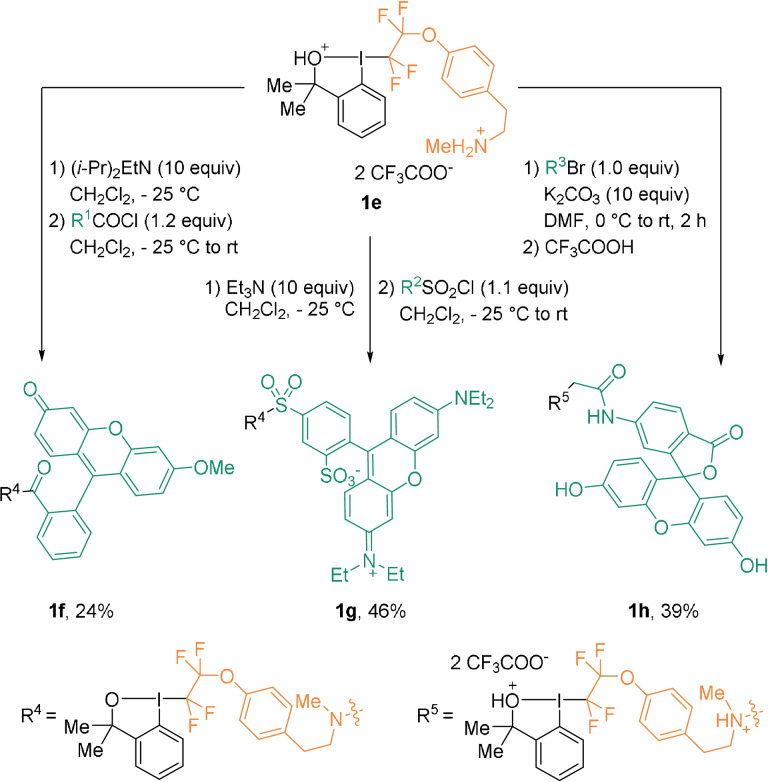
Selected examples of the derivatization of reagent **1 e**.

As **1 c** and **1 d** required a multistep synthesis, a modular reagent **1 e** was developed as a precursor to a wide range of bioconjugation reagents (Scheme 4).^[17a]^ **1 e** features a secondary amine that can be involved in classical linking methods compatible with the hypervalent iodine moiety. For example, amide, sulfonamide, or tertiary amine bonds can be easily generated, yielding reagents **1 f**–**h**.

**Scheme 5:**
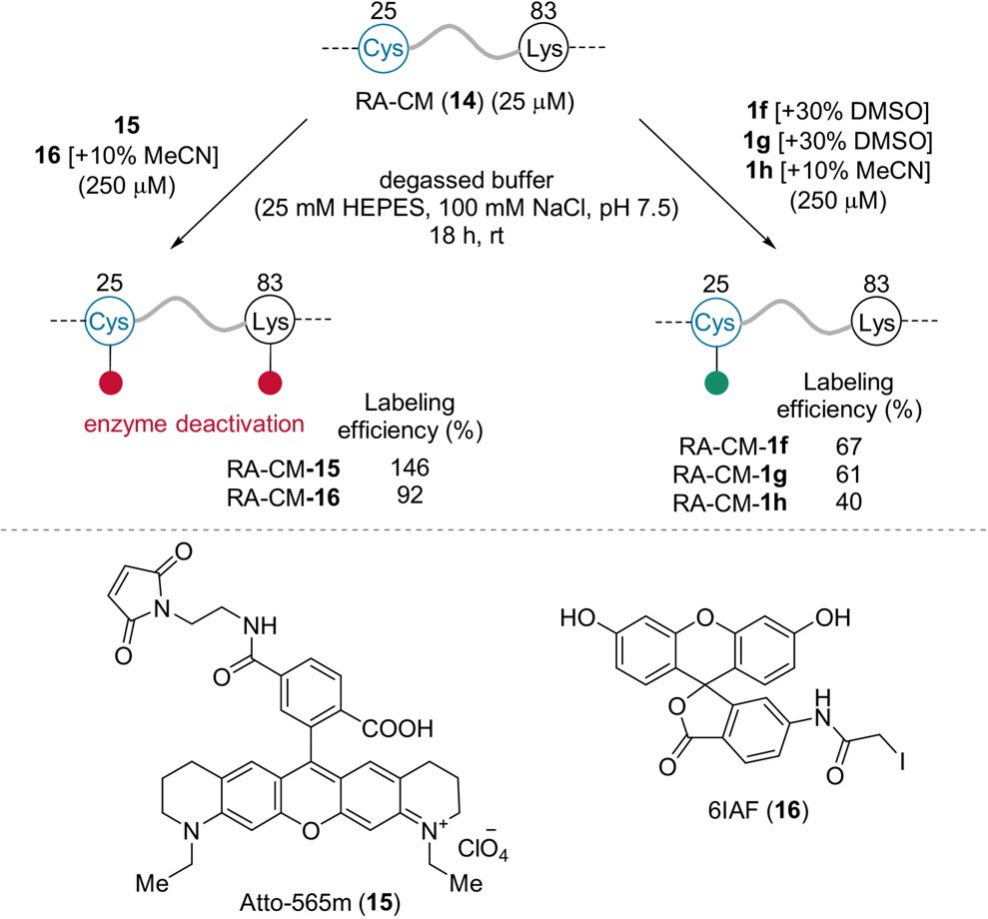
The functionalization of RA‐CM (**14**). Labeling efficiency: average percentage of chromophores attached to one protein.

Reagents **1 f**–**h** were examined for labeling the laboratory‐evolved retro‐aldolase RA95.5‐8 S25C K210M (RA‐CM, **14**), which contains an exposed cysteine (Cys25) and a lysine (Lys83) residue (Scheme 5).^[17a]^ The commercially available fluorescent probes Atto‐565‐maleimide (Atto‐565m, **15**) and 6‐(iodoacetamido)fluorescein (6IAF, **16**) were shown to modify not only Cys25, but also Lys83. This deactivates the enzyme, since Lys83 is essential for its catalytic activity. In contrast, **1 f**–**h** exclusively labeled Cys25. The degree of labeling of the new reagents exceeded the level reported with sub‐stoichiometric quantities of Atto‐565m (which were used to avoid undesired reactions on Lys83).^[18]^

**Scheme 6:**
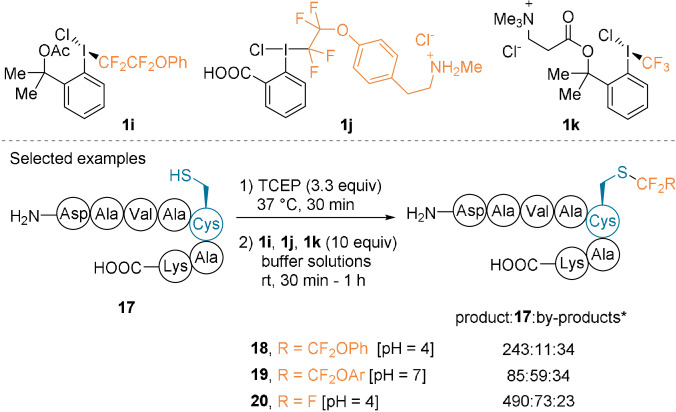
Noncyclic hypervalent iodine reagents **1 i**–**k** and fluoroalkylation of **17**. * Area under the curve (AUC×10^2^) determined by the integration of mass ion chromatograms measured on the crude mixture. By‐products result from sulfur oxidation.

Thereafter, the Beier group described new water‐soluble noncyclic reagents, such as **1 i**–**k** (Scheme 6).^[19]^ Despite their poor stability in aqueous media, they efficiently converted heptapeptide **17** into the fluoroalkylated products **18**–**20** in acidic (pH 4) or neutral (pH 7) buffers. Although the reaction was selective for the Cys residues, various sulfur‐oxidized side products were detected due to the excess reagent used.

**Scheme 7:**
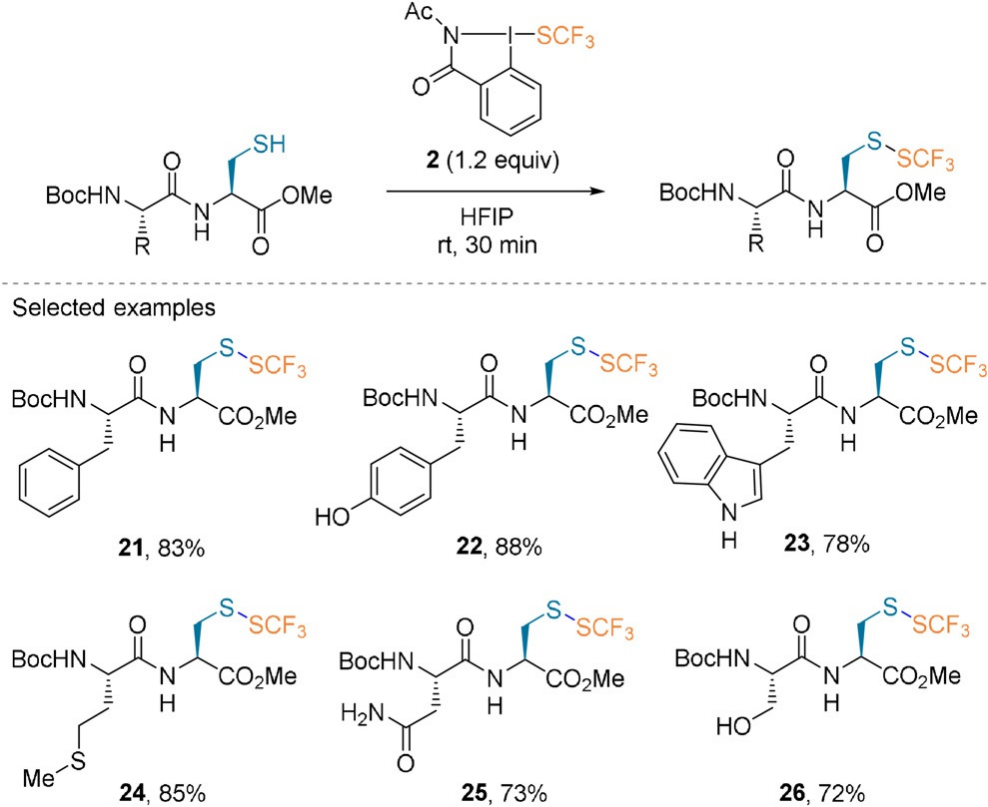
Trifluoromethylthiolation of dipeptides.

In addition to the studies by the Togni and Beier groups on fluoroalkylation, Zhang and co‐workers described the synthesis of the trifluoromethylthiolating reagent **2** based on an *N*‐acetylbenziodazole core.^[9]^ Cys‐containing dipeptides were converted in high yields and chemoselectivity in 30 minutes into disulfides **21**–**26** (Scheme 7).

#### 2.1.2 Alkynylation of Cys

Alkynes are desired reactive handles and, therefore, are often incorporated onto biomolecules. In particular, terminal alkynes have been introduced, as they can undergo CuAAC (copper(I)‐catalyzed alkyne‐azide cycloaddition), Glaser‐Hay coupling, Ru^II^‐catalyzed alkyne hydrosilylation as well as thiol‐yne reactions.^[20]^

**Scheme 8:**
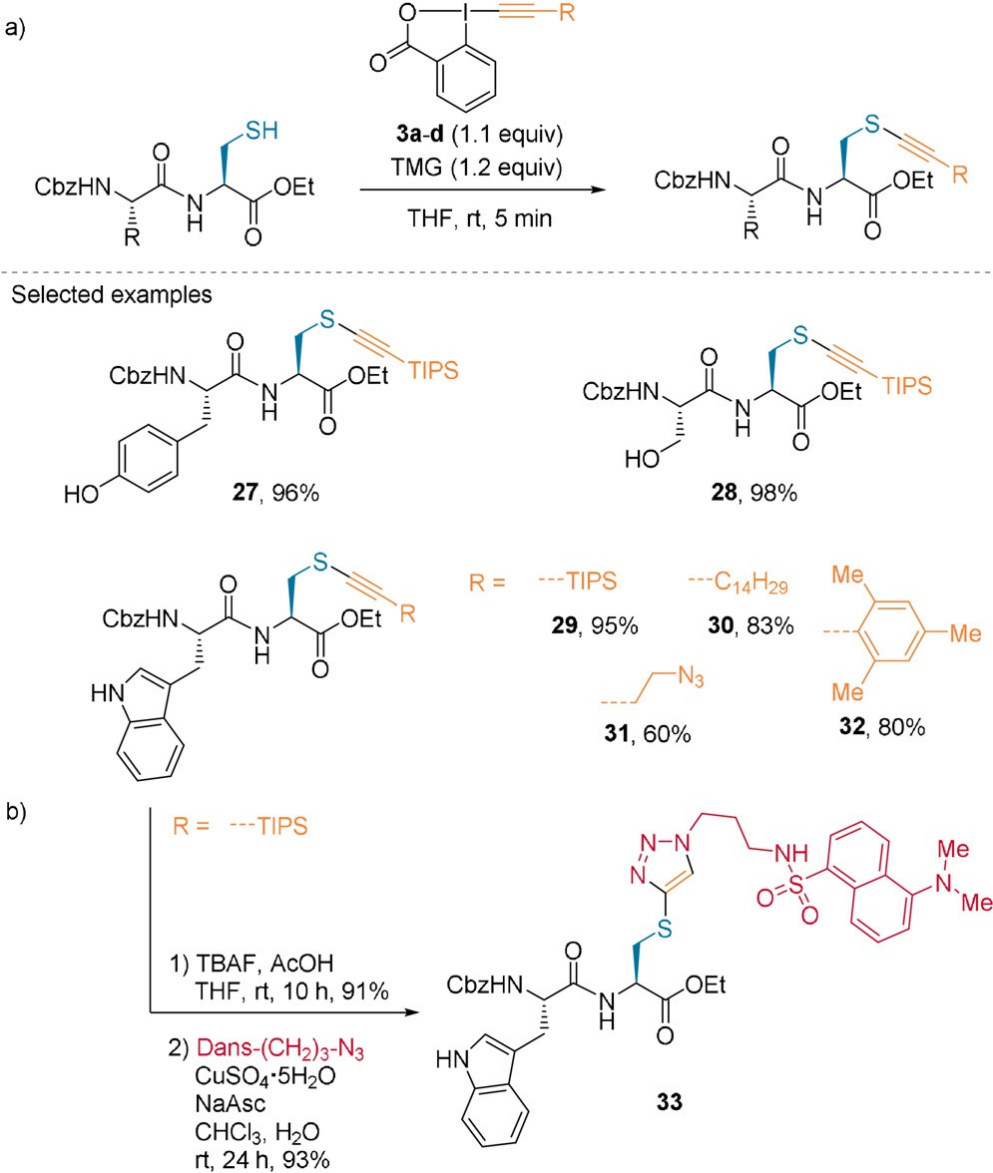
a) Alkynylation of Cys‐containing dipeptides. b) Functionalization of the thioalkyne product **29** by CuAAC.

In 2013, our research group applied the commercially available hypervalent iodine reagent 1‐[(triisopropylsilyl)ethynyl]‐1,2‐benziodoxol‐3(1*H*)‐one (TIPS‐EBX, **3 a**) to various Cys‐containing dipeptides.^[10]^ Under mild reaction conditions, thioalkynes **27**–**29** were obtained in excellent yields and chemoselectivity (Scheme 8 a). In addition to the TIPS group, alkynes bearing various substituents, such as a long alkyl chain (**30**), an alkyl chain bearing an azide (**31**), and a hindered aromatic group (**32**) were successfully introduced on a dipeptide.^[21a]^ Moreover, a fluorophore tag was added in a two‐step process: removal of the TIPS group and subsequent CuAAC to give **33** (Scheme 8 b).^[10]^

**Scheme 9:**
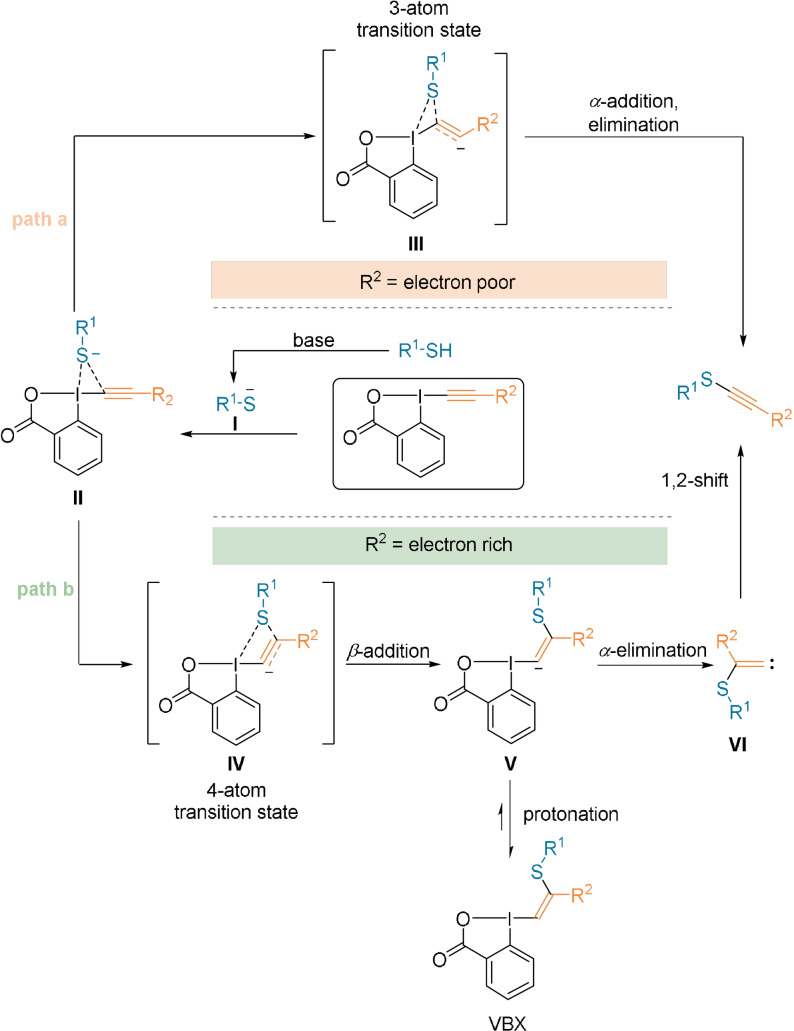
Proposed mechanistic pathways for thioalkynylation using R‐EBX reagents. R^2^=OMe, Me, SiMe_3_, Si*i*Pr_3_, Ph, CO_2_Me.

The reaction mechanism was investigated using experimental and computational methods.^[21]^ The first step is believed to be the formation of the thiolate **I**, since in the absence of a base, only traces of the thioalkyne product were observed (Scheme 9). **I** can then interact with the Lewis‐acidic iodine (**II**). From here, there are two possible reaction pathways. In the case of α‐addition of the thiolate **I**, computational studies revealed low energy transition state **III** (path a). **III** corresponds to a concerted α‐addition/elimination to give the thioalkyne product. In contrast, attack of thiolate **I** on the β‐carbon atom occurs via the transition state **IV** (path b) leading to β‐addition and formation of the vinyl anion **V**. The latter then undergoes an α‐elimination to form **VI**, which, after a 1,2‐shift, yields the thioalkyne. Alternatively, in protic solvents, intermediate **V** can be protonated, leading to vinylbenziodoxolone (VBX) products.

Calculating the free energy values of the transition states for different hypervalent iodine reagents revealed that the R^2^ group on the alkyne has a strong influence on the reaction pathway. When R^2^ is an electron‐withdrawing group, the route going through an α‐addition is the lower energy pathway, since the partial negative charge is stabilized. In contrast, when R^2^ is an electron‐donating group, such as an alkyl substituent, the preferred pathway is the β‐addition. For aryl and silyl substituents, both pathways are possible, since the energy difference is small.

**Scheme 10:**
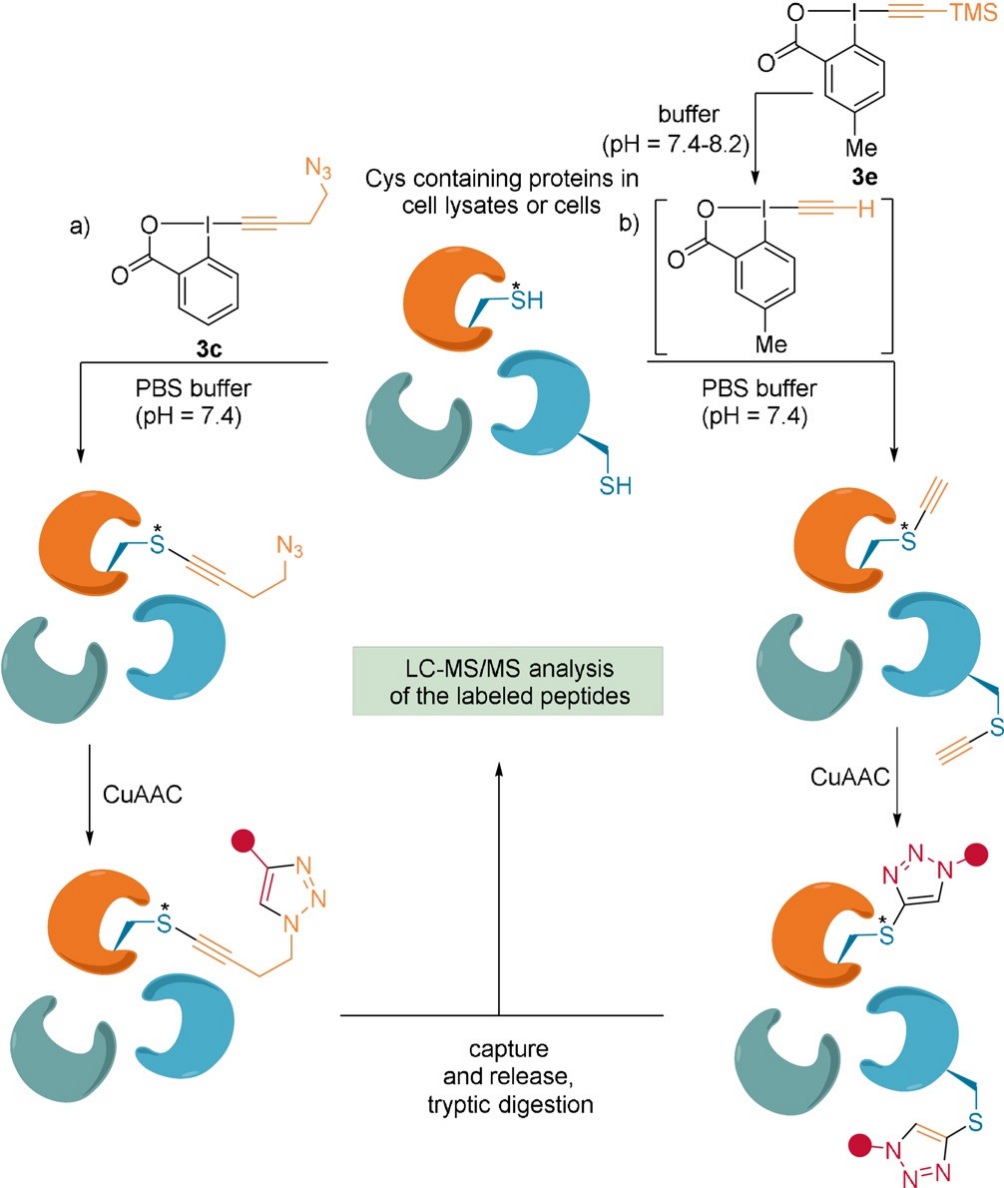
Proteomic target profiling using EBX reagents a) **3 c** and b) **3 e** in HeLa cell lysates. Hyperreactive Cys is noted with *.

In joint efforts by Adibekian's and our group, the thioalkynylation methodology was applied to in vitro and in situ proteomic profiling of Cys residues.^[22]^ To avoid the need for cleavage of the TIPS group, but still allow functionalization of proteins through a CuAAC reaction, the EBX reagent **3 c**, carrying an azide, was used (Scheme 10 a).

The reactivity of **3 c** was tested on purified catalase and complete cell lysates under physiological conditions. Under these neutral conditions, only more acidic—hyperreactive—Cys residues were deprotonated and reacted. It is believed that, due to the high reactivity and lipophilic environment of the Cys residues, induced by neighboring amino acids, thioalkynes rather than VBXs were obtained as major products, whereas the reverse is observed for Cys with regular reactivity (see Section 2.1.3.).^[23]^ **3 c** displayed higher efficiency, lower cytotoxicity, and higher chemoselectivity compared with the commonly used Cys probe iodoacetamide (IAA) or IAA‐alkyne. Reagent **3 c** was used for target discovery of the anticancer and anti‐inflammatory agent curcumin. It was demonstrated to be complementary to IAA‐alkyne, since out of the 42 detected targets, 16 were exclusively modified by **3 c**.

In 2020, our group developed reagents bearing a TMS group and various aromatic substituents, which under aqueous media undergo fast TMS cleavage (Scheme 10 b).^[24]^ For example, reagent **3 e** provided improved Cys labeling in HeLa cell lysates compared to **3 c** (Scheme 10). After digestion of the labeled proteins, 4325 labeled peptides were detected for **3 e**, while 2257 were observed for **3 c**. The terminal thioalkynes were formed as the only products after rearrangement of the VBX intermediates with high Cys selectivity. The method was also extended to in situ labeling of living HeLa cells.

**Scheme 11:**
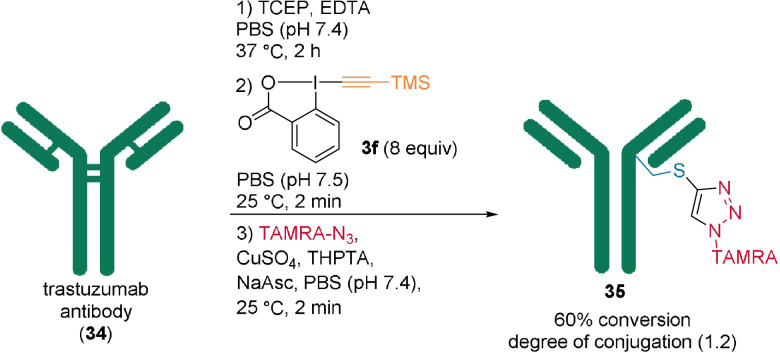
Use of TMS‐EBX (**3 f**) for the functionalization of antibody **34**.

Next, modification of an antibody approved for breast cancer treatment, trastuzumab (**34**), was explored. The use of 8 equivalents of **3 f** at pH 7.5 and 25 °C for 2 minutes (Scheme 11) gave 60 % conversion of the reduced trastuzumab (**34**) to yield TAMRA‐labeled **35** with an average degree of conjugation (DoC) value of 1.2 after CuAAC. Under these conditions no side‐reactivity was observed, also with non‐reduced trastuzumab. Varying the reaction parameters led to DoC values ranging from 0.1 to 4.4. Whereas some side reactivity with the non‐reduced antibody was observed under most conditions, it was outcompeted by cysteine alkynylation of the reduced antibody.

**Scheme 12:**
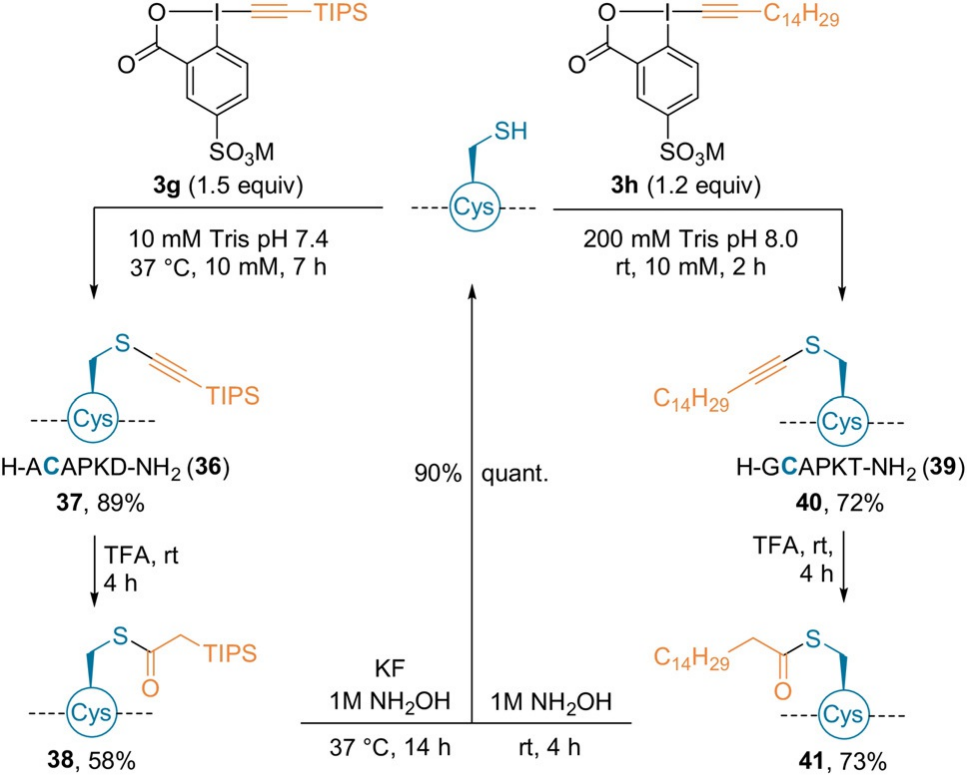
Lipidation of hexapeptides using reagents **3 g** and **3 h**. M=Na or K.

**Scheme 13:**
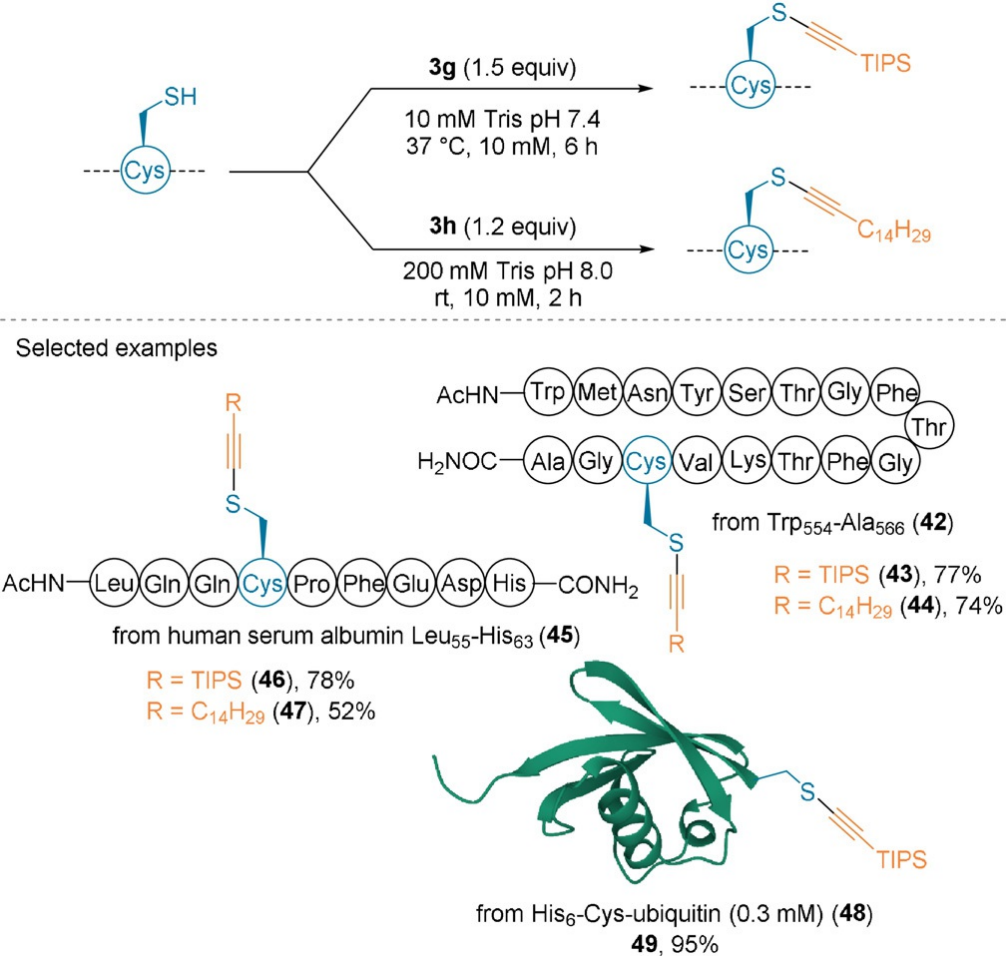
Lipidation of longer peptides and a protein using **3 g** and **3 h**.

Later, amphiphilic hypervalent iodine reagents were introduced for the lipidation of Cys residues.^[25]^ Reagents bearing a TIPS group (**3 g**) or a C_14_ alkyl chain (**3 h**) were synthesized (Scheme 12). Water solubility was achieved by introduction of a *p*‐sulfonate group on the aromatic core. The presence of the sulfonate group led to thioalkynes as the major products, even with alkyl substituents. Both reagents were applied to a variety of hexapeptides, and yielded, for example, **37** and **40** in yields of 89 and 72 % in aqueous buffers. Reversibility of the functionalization was achieved by hydration of the thioalkyne (**38**, **41**) and subsequent cleavage with hydroxylamine. The method was further extended to longer peptides **42** and **45**, as well as to a His6‐Cys‐ubiquitin protein **48** at micromolar concentration (Scheme 13).

**Scheme 14:**
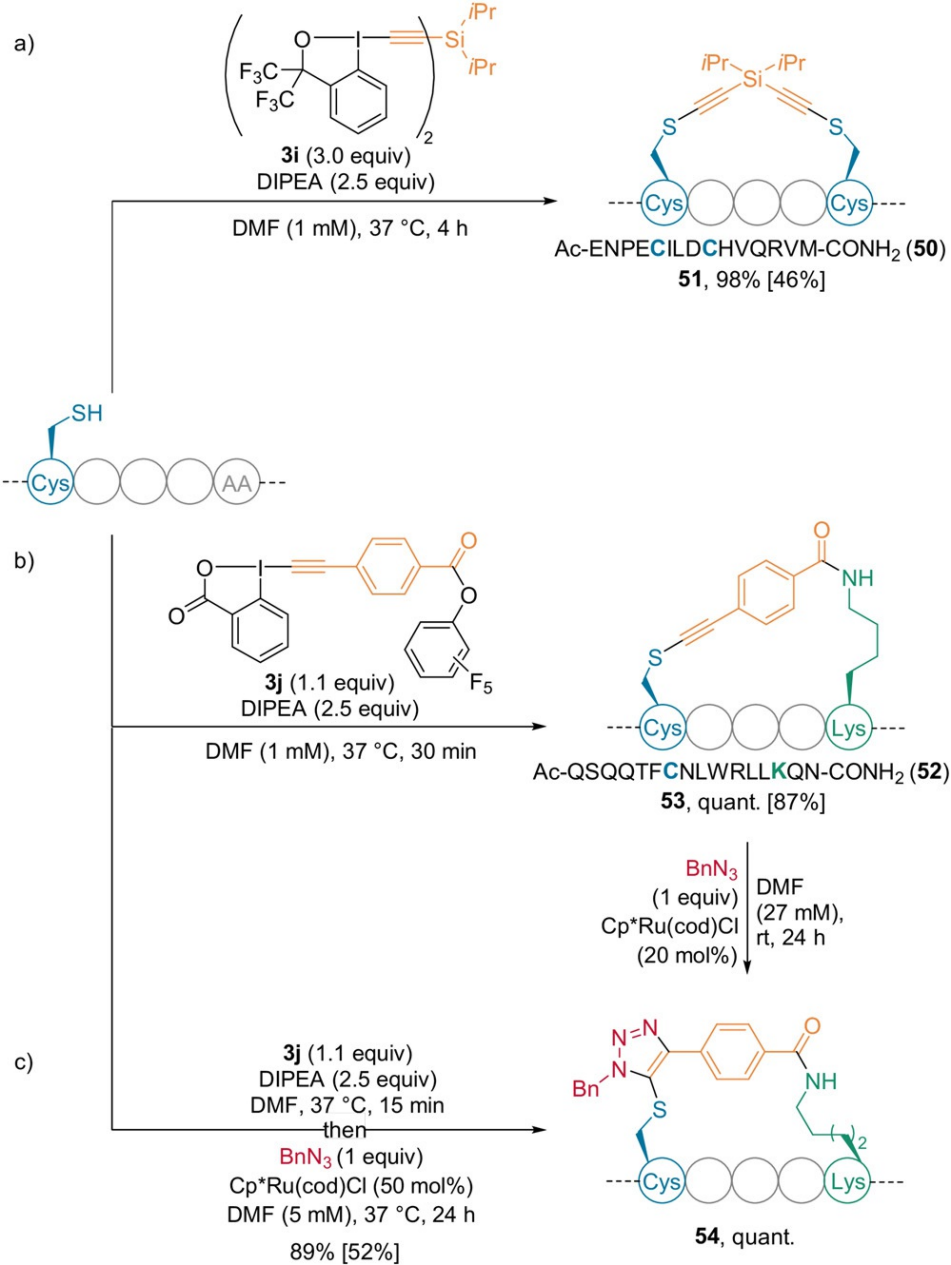
Selected examples of a) Cyc‐Cys stapling, b) Cys‐Lys stapling and the subsequent click reaction. c) One‐pot Cys‐Lys stapling followed by a click reaction. Yields of isolated products are given in brackets.

In 2020, doubly functionalized reagents were developed for peptide stapling.^[26]^ Bis‐hypervalent iodine reagents, linked by phenyl or various silicon groups, were synthesized with Cys cross‐linking. In particular, reagent **3 i** provided the *i*,*i*+4 stapled peptide **51** in high yield (Scheme 14 a).

Installation of an activated ester allowed Cys‐Lys stapling to be performed. Reagents bearing phenyl linkers with various substitution patterns were reported. For example, excellent reactivity was observed with the *para*‐substituted reagent **3 j** (Scheme 14 b), particularly when applied for *i*,*i*+7 stapling of peptide **52** derived from p53 protein. The obtained product **53** showed increased helicity and binding affinity to MDM2 protein—a native binder of p53 protein and a known cancer target. Interestingly, since the reaction with Cys occurs first, proximity‐driven Lys selectivity was observed. A post‐stapling modification for the Cys‐Lys cross‐linked products was achieved by performing RuAAC with the thioalkyne present on the linker. The triazole product **54** was generated in high yield and regioselectivity using either isolated stapled product (Scheme 14 b) or in a one‐pot manner (Scheme 14 c).

#### 2.1.3 Vinylation of Cys

**Scheme 15:**
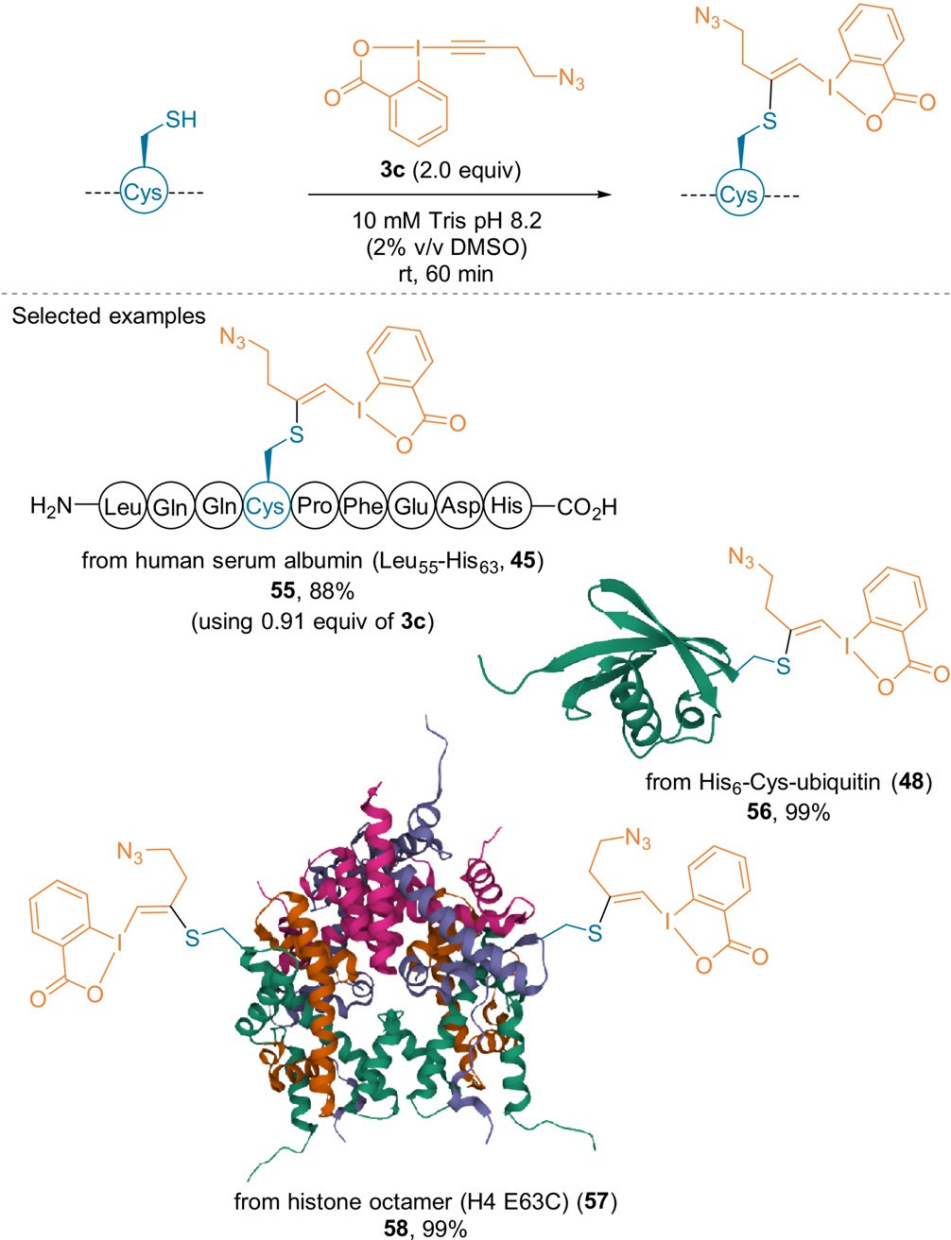
VBX formation on a peptide and proteins using **3 c**.

In 2019, the application of the reagent **3 c** was extended from hyperreactive Cys (Scheme 10 a) to the less acidic surface‐exposed Cys.^[23]^ A basic buffer (pH 8.2) was used to efficiently convert less‐reactive Cys residues, thereby leading to VBX products (Scheme 15). **3 c** was applied to the human serum albumin Leu55–His63 sequence (**45**) as well as the modified proteins His6‐Cys‐ubiquitin (**48**) and E63C‐histone octamer (**57**), which exclusively provided the VBX products **55**, **56,** and **58**.

**Scheme 16:**
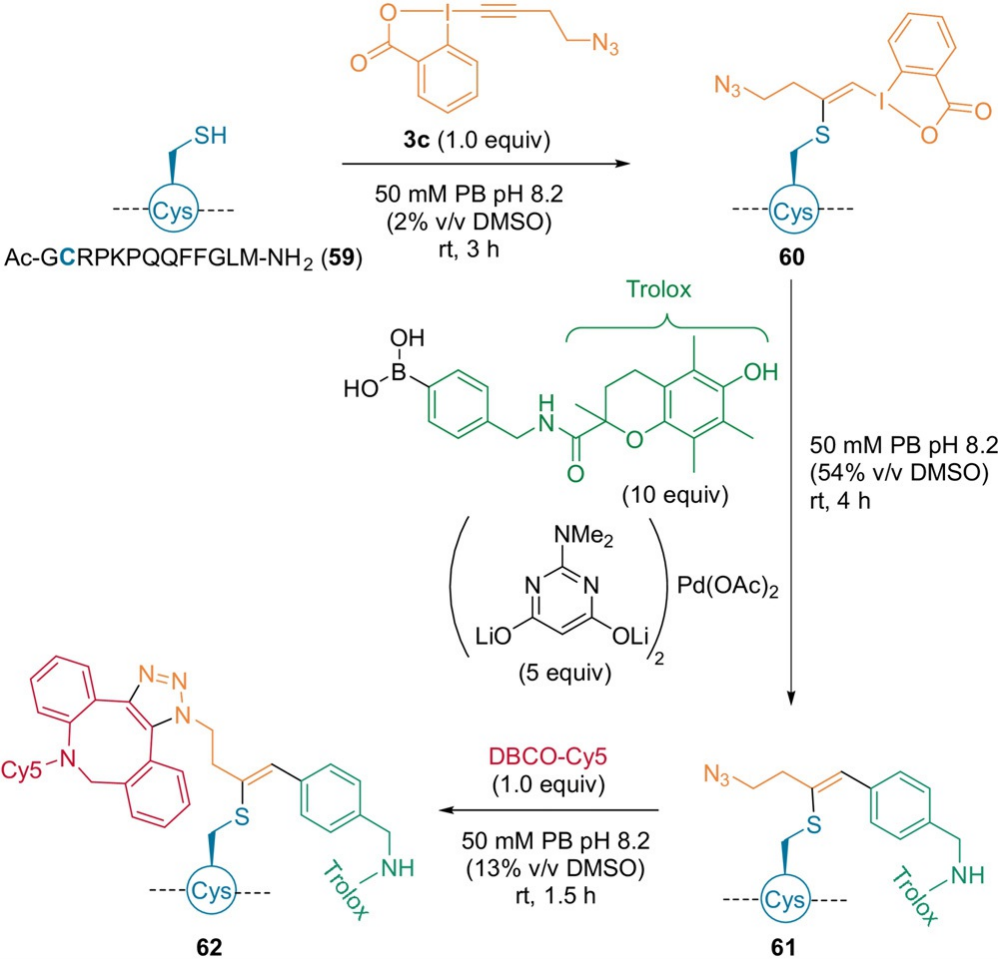
Double functionalization of VBX product **60**.

The presence of a hypervalent moiety enabled an additional modification, as demonstrated with the functionalized neuropeptide **60** (Scheme 16). The photoprotection compound Trolox was introduced through a Suzuki coupling between a boronic acid and the VBX moiety on **60** to give **61**. Then a Cy5 dye was added using strain‐promoted cycloaddition to afford **62**. The bleaching time of the dye was increased by a factor of 3 compared to when the Trolox moiety was absent.

### 2.2 Methionine

Methionine (Met) has an important role in many biological processes. However, as a result of its low nucleophilicity and high hydrophobicity, it has been modified less often than Cys.^[27]^ Nevertheless, high Met selectivity can be achieved under acidic conditions, thus making it useful for the development of site‐selective bioconjugation methods.^[28]^

**Scheme 17:**
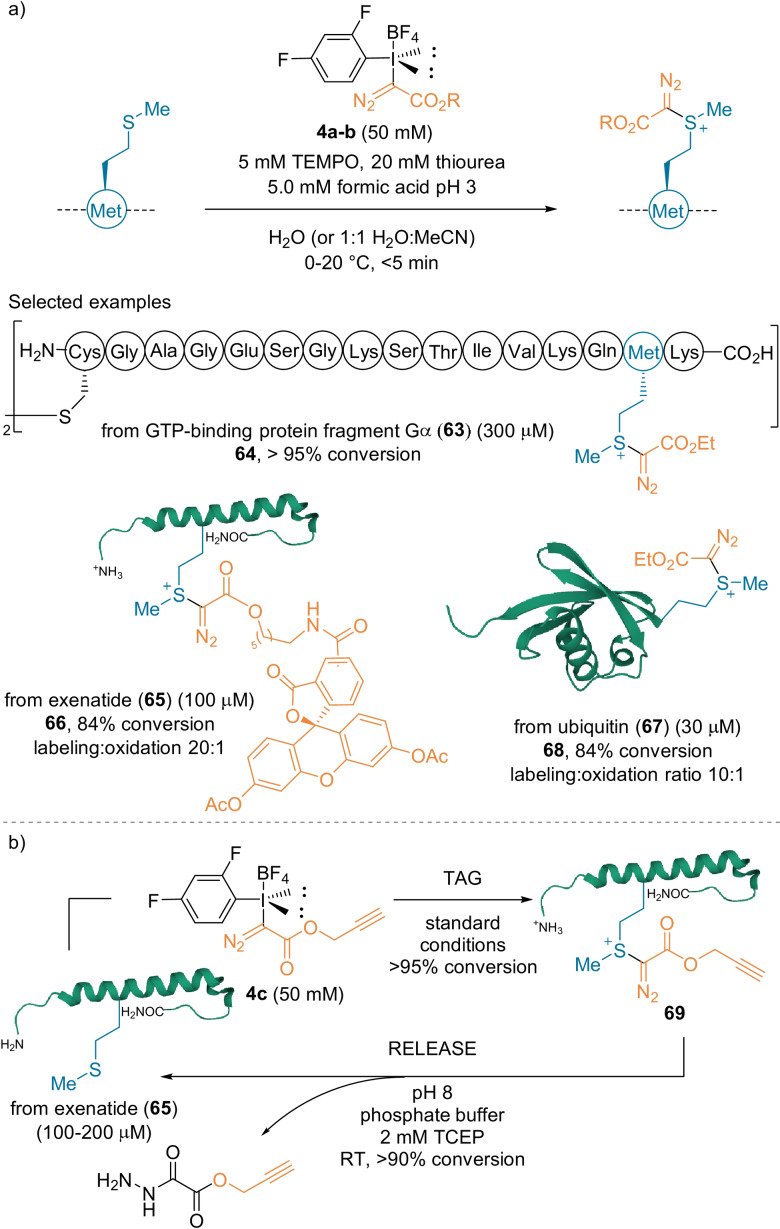
Met‐selective bioconjugation of peptides and proteins.

The Gaunt group used the fine‐tuned acyclic hypervalent iodine reagents **4 a**–**c** for peptide and protein labeling at Met residues (Scheme 17).^[29]^ These types of compounds have been reported as efficient electrophilic diazo transfer reagents,^[30]^ and allowing the generation of high‐energy sulfonium conjugates. Additives, such as thiourea, TEMPO, and formic acid (pH≈3) were required to minimize decomposition of the starting biomolecules, oxidized side‐product formation, and achieve high selectivity. The authors established in one case that the helical structure of the peptide was conserved after labeling, but no extensive studies were performed on the structural integrity and activity of other conjugates. The reaction was tested on several peptides and proteins using diazo motifs bearing different substituents. High conversions were obtained in less than five minutes. Disulfide linkages and a fluorescein‐derived ester were tolerated (**64**, **66**). As the N‐terminal Met residue has only moderate surface exposure in ubiquitin **67**, the reaction was performed under deoxygenated conditions to minimize competitive oxidation. High conversion into the labeled product **68** with a good 10:1 labeling/oxidation ratio was obtained. As demonstrated with exenatide **65**, reversibility and an excellent conversion was achieved using the tertiary phosphine tris(2‐carboxyethyl)phosphine (TCEP, Scheme 17 b).^[31]^

**Scheme 18:**
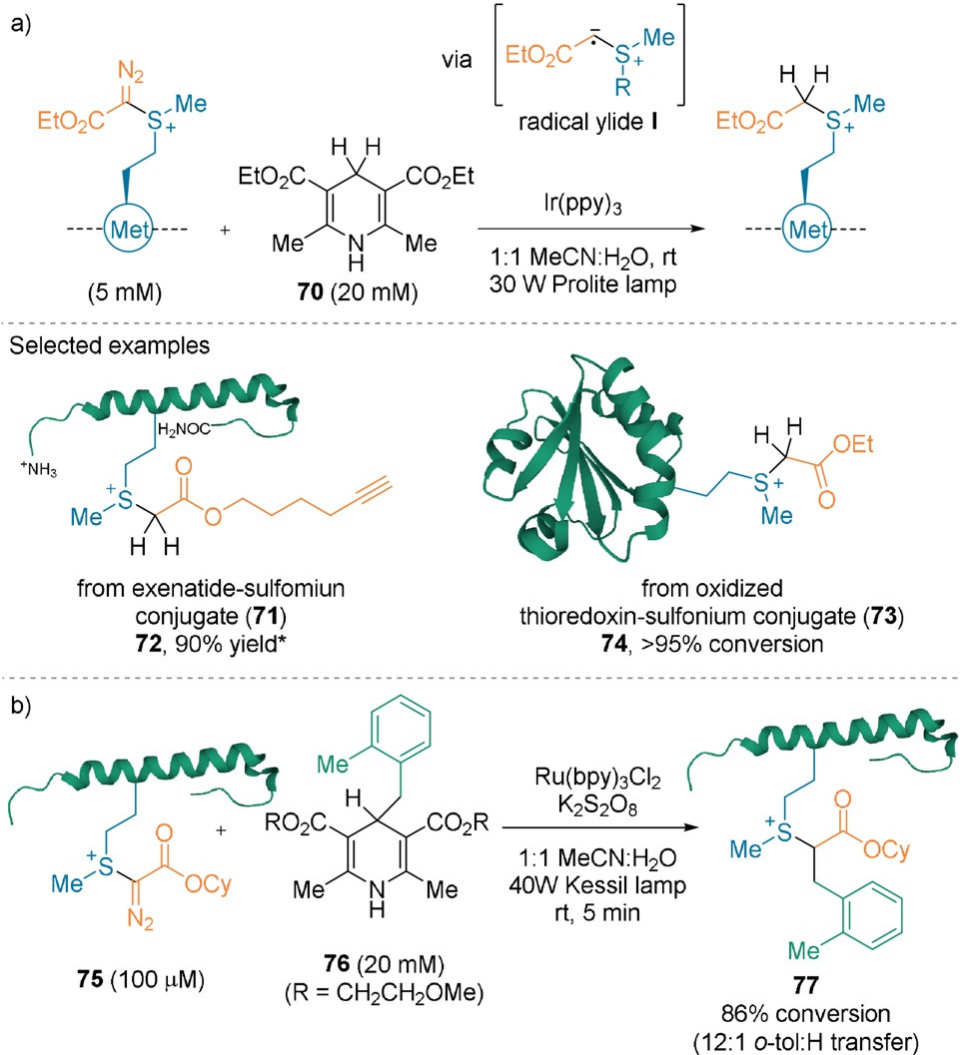
Photoredox‐mediated a) reduction of sulfonium conjugates and b) radical cross‐coupling. * Determined by ^1^H NMR spectroscopy.

The sulfonium products are stable enough for further derivatization. Irradiation led to the formation of radical ylides **I**, which can react with Hantzsch esters (Scheme 18).^[32]^ Reduced trialkylsulfonium motifs **72** and **74** were obtained with high conversions using **70**, without affecting the disulfide bridge of **73** (Scheme 18 a). **77** was formed with high conversion when the C4‐benzylated Hantzsch ester **76** was used (Scheme 18 b). It is noteworthy that the Met bioconjugation and photoreduction steps can be carried out in a one‐pot procedure, without compromising the yield or purity of the products.

## 3 Aromatic Amino Acids

Although Cys bioconjugation is broadly applied, this residue is usually in the form of disulfide bridges or is an essential component of the active site of natural enzymes. Therefore, modification of other amino acids is also attractive. For example, the functionalization of tryptophan (Trp)^[33]^ and tyrosine (Tyr)^[34]^ can provide site‐specific modifications, as Trp is the rarest amino acid and Tyr is rarely exposed on the surface.

### 3.1 Tryptophan

#### 3.1.1 Fluoroalkylation of Trp

**Scheme 19:**
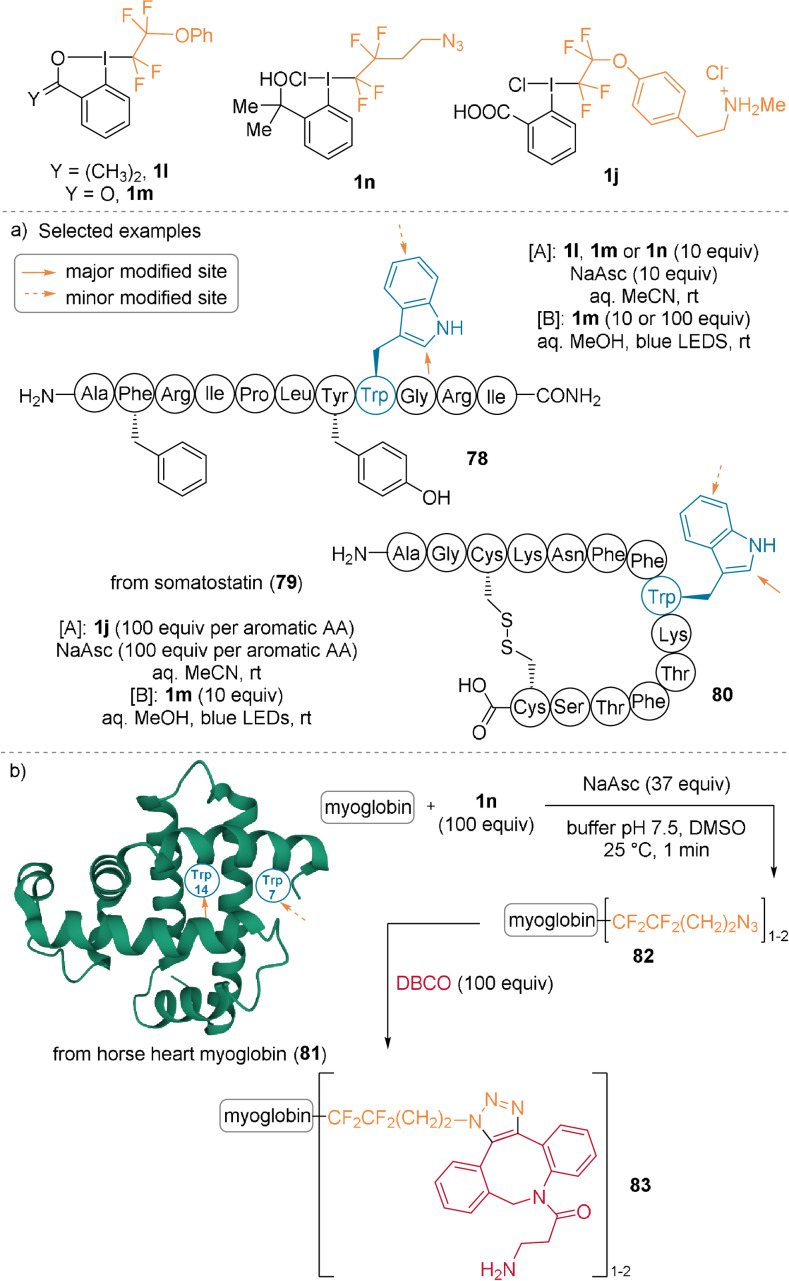
a) Fluoroalkylation of Trp‐containing peptides using conditions A or B. b) Labeling of myoglobin.

Functionalized tetrafluorinated hypervalent iodine reagents were used for the modification of Trp residues in peptides and proteins. The Novák and Beier groups discovered that sodium ascorbate triggers the generation of tetrafluoroethyl radicals and their reaction with Trp residues (Scheme 19, conditions A).^[35]^ Later, visible light was used as the radical initiator (conditions B).^[36]^ Preference for the Trp C2‐position was observed, but modification of the phenyl ring also occurred. Tolerance to a range of functional groups, including aromatic and nucleophilic residues, as well as a disulfide bridge, was observed (**78**, **80**). However, when the reaction was applied to a peptide containing a free Cys, both the Trp and Cys residues were functionalized.^[36]^ The sodium ascorbate initiated reaction was applied to myoglobin **81** using azide‐substituted reagent **1 n** (Scheme 19 b). The more exposed Trp14 was primarily functionalized, while Trp7 was modified to a lesser degree. A cycloaddition reaction with dibenzocyclooctyne‐amine (DBCO‐amine) was performed, which afforded labeled protein **83**.

#### 3.1.2 Alkynylation of Trp

**Scheme 20:**
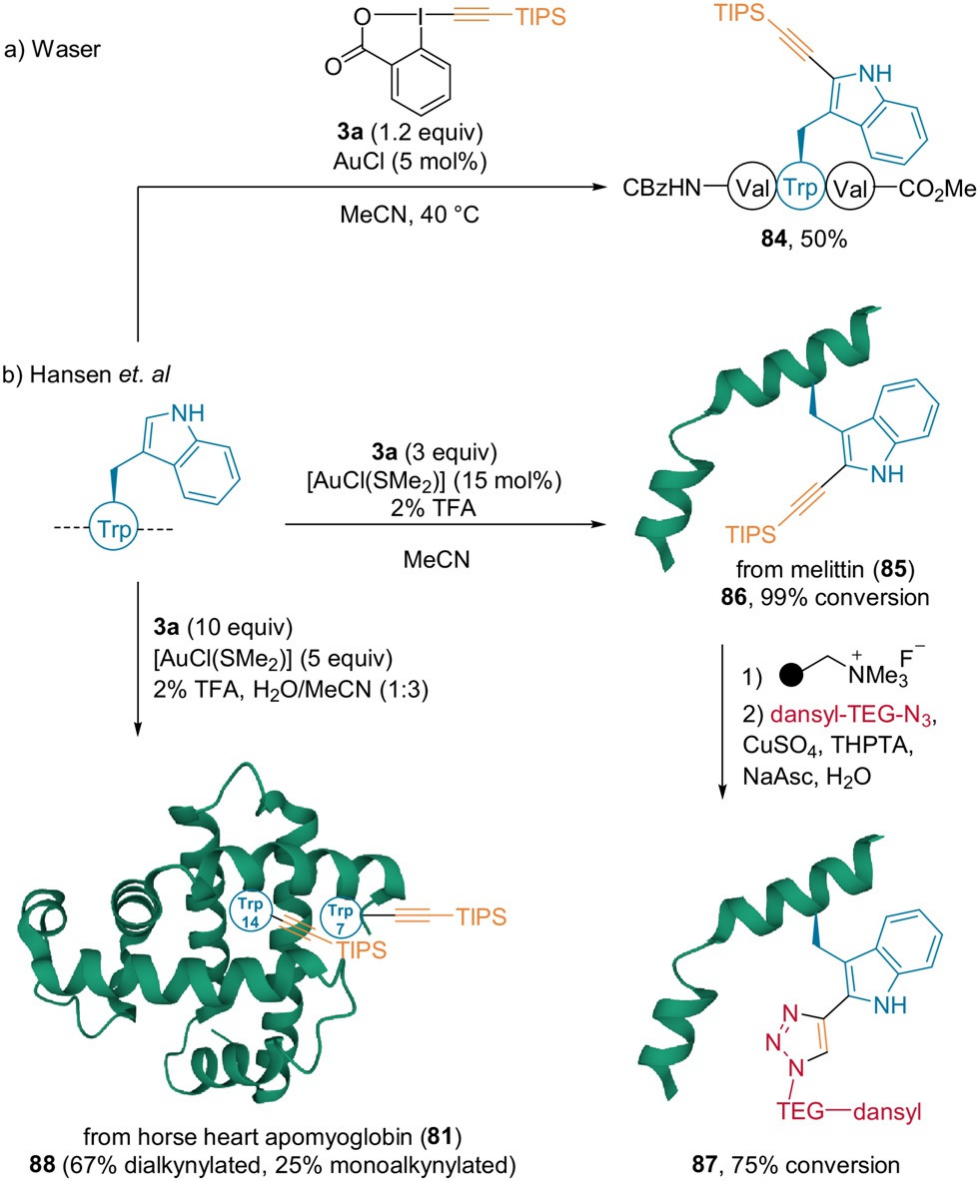
Selected examples of Trp‐alkynylation using TIPS‐EBX (**3 a**).

In 2009, our group described the gold‐catalyzed alkynylation of the C3‐position of indoles, when the C2‐position was blocked, using TIPS‐EBX **3 a**.^[37]^ The exact mechanism of this reaction is still unknown, but has been proposed to involve alkyne transfer from iodine to gold without oxidative addition.^[38]^ In 2016, the scope of the reaction was extended to Trp in independent studies by our group^[39]^ and Hansen et al. (Scheme 20).^[40]^ Using AuCl in acetonitrile, our group applied the method up to tripeptides, thereby providing the desired alkynylated product **84** in moderate yield (Scheme 20 a). Hansen et al. demonstrated that the reactivity can be improved by the addition of a weakly coordinating ligand (Me_2_S) and 2 % TFA.^[40]^ Full conversion was achieved with melittin **85**—a peptide consisting of 26 amino acids, including Lys, Thr, Arg, and Ser, using a catalytic amount of gold (**86**, Scheme 20 b). The alkyne‐TIPS group was removed to perform a click reaction with dansyl‐TEG‐azide, thereby yielding fluorescent‐labeled melittin **87**. The method was also applied to apomyoglobin **81**, although a significant amount of acetonitrile and 5 equivalents of the gold complex were needed to achieve a good conversion and to obtain 67 % of the dialkynylated and 25 % of the monoalkynylated products **88**.

#### 3.1.3 Arylation of Trp

**Scheme 21:**
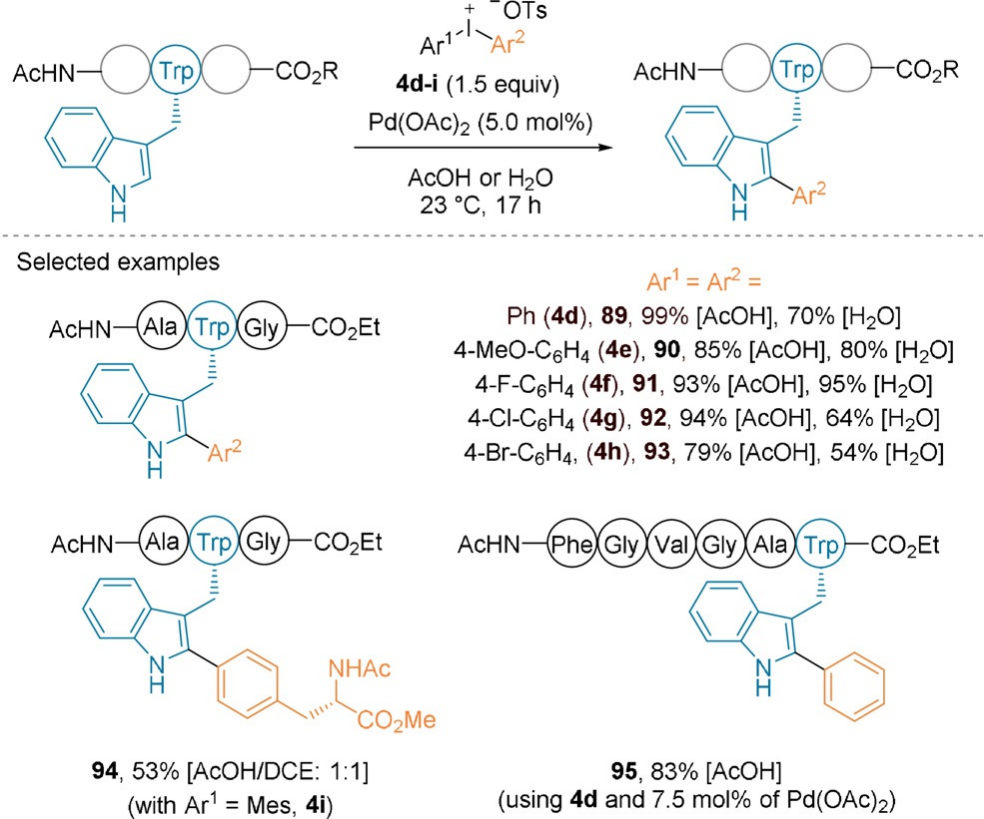
C−H arylation of Trp‐containing peptides using diaryliodonium salts **4 d**–**i**.

In metal‐catalyzed reactions, diaryliodonium salts behave as more reactive versions of aryl halides.^[41]^ Using symmetrical or unsymmetrical reagents **4 d**–**i**, the Ackermann group developed a late‐stage peptide C−H arylation of the C2‐position of Trp residues (Scheme 21).^[42]^ Peptides (up to 6 amino acids long) were arylated in good to excellent yields at ambient temperature using 5 mol % Pd(OAc)_2_ and acetic acid or pure water as the solvent to give **89**–**95**. The reaction tolerated other aromatic residues, but protecting groups on the nucleophilic ones were required. An unusual peptide coupling was performed using an asymmetric iodonium reagent bearing a phenylalanine residue to give **94**.

### 3.2 Tyrosine

**Scheme 22:**
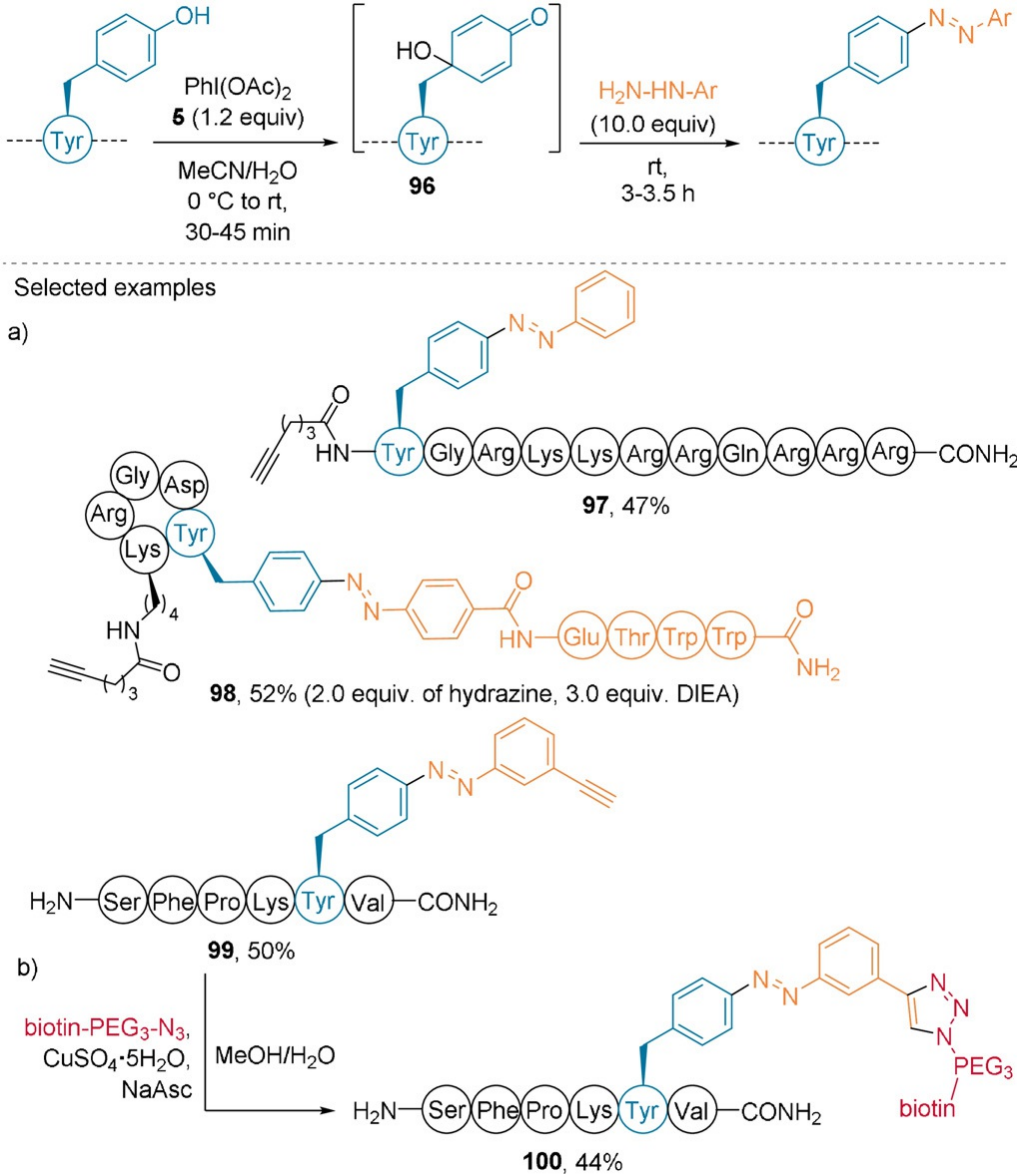
a) Tyr oxidation using PhI(OAc)_2_ (**5**) and functionalization using a hydrazine. b) CuAAC functionalization of **99**.

Recently, Wang et al. used (diacetoxyiodo)benzene (PIDA, **5**) for the oxidation of phenol in Tyr residues, through formation of the reactive intermediate 4‐hydroxycyclohexadienone **96** (Scheme 22).^[43]^ Subsequent reaction with arylhydrazine yielded *trans* isomers of azobenzene‐functionalized peptides. The reaction was applied to a range of unprotected peptides containing up to 11 amino acids to give azobenzenes such as **97**–**99**, thus demonstrating broad functional group tolerance (Scheme 22 a). In addition, functionalized hydrazines were successfully introduced. A hydrazine‐substituted peptide was utilized, which yielded **98**. Reaction with a clickable alkyne moiety resulted in the biotin‐functionalized peptide **100** after CuAAC (Scheme 22 b).

The oxidation of Tyr residues using PIDA (**5**) has been also used for intramolecular coupling with Trp to synthesize natural products.^[44]^

## 4 Aliphatic Amino Acids

The late‐stage derivatization of peptides and proteins targeting non‐activated C−H bonds is challenging due to their low reactivity and the difficulty of achieving selectivity. Nevertheless, highly reactive hypervalent iodine reagents have been successfully applied for the functionalization of leucine (Leu) and alanine (Ala) residues.

### 4.1 Leucine

**Scheme 23:**
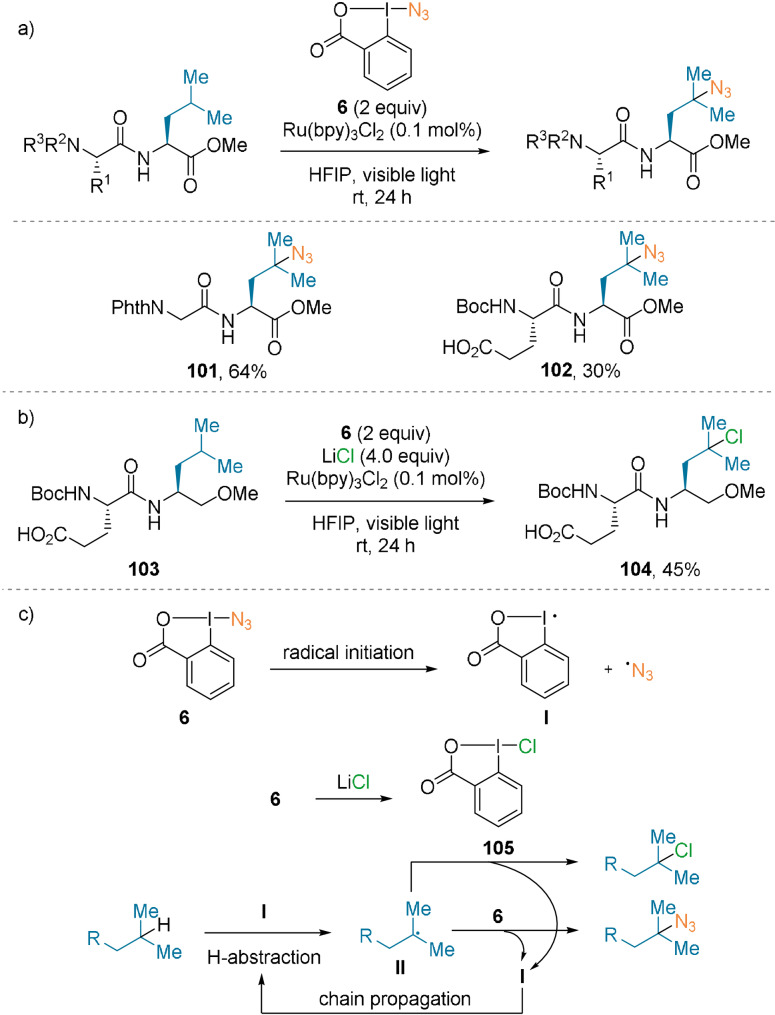
C(sp^3^)−H a) azidation and b) chlorination of dipeptides. c) Plausible mechanism.

Chen and co‐workers described an azidation method that targeted tertiary C−H bonds.^[45]^ By using the Zhdankin reagent 1‐azido‐1,2‐benziodoxole‐3‐(1*H*)‐one (ABX, **6**),^[46]^ visible light, and Ru(bpy)Cl_2_ as the photosensitizer, C‐terminal Leu residues in dipeptides were functionalized in moderate yields (**101** and **102**, Scheme 23 a). Excellent selectivity for the tertiary C−H bond was observed. Interestingly, the use of lithium chloride as an additive led to C−H chlorination, thereby yielding dipeptide **104** in 45 % yield (Scheme 23 b).

The reaction is believed to be initiated by a homolytic cleavage of the weak I−N_3_ bond of **6**, thereby generating azido and iodanyl **I** radicals (Scheme 23 c). Then, **I** can perform a hydrogen abstraction on the substrate to yield intermediate **II**. This radical can then attack reagent **6** to form the C−N_3_ bond, regenerating **I**, and propagating the radical chain. Interestingly, the chlorination reaction seems to go through the same initiating step. The chlorinated hypervalent analogue **105** is believed to be generated in situ by an exchange of azide with chloride. However, the reaction did not proceed when only the isolated reagent **105** was used, since the I−Cl bond is harder to cleave. The ABX reagent **6** is required for radical chain initiation. **105** is, however, more reactive than **6** towards nucleophilic attack of the C‐tertiary radical **II**, and generates the chlorinated products.

**Scheme 24:**
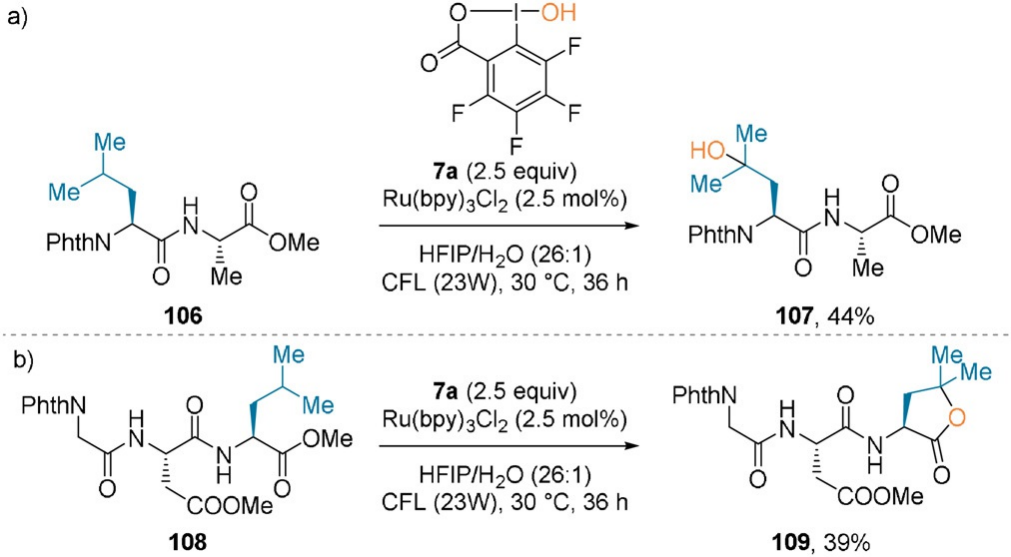
C(sp^3^)−H hydroxylation of a Leu‐containing a) dipeptide and b) tripeptide. CFL=household compact fluorescent lamp.

The same research group later described a C(sp^3^)−H hydroxylation method.^[47]^ Hydroxyperfluorobenziodoxole (PFBI‐OH, **7 a**) was used as it has a strong H‐abstraction ability. The reaction proceeded under similar photocatalyzed conditions with excellent selectivity. Hydroxylated dipeptide **107** was isolated in 44 % yield, while lactone formation was observed with C‐terminal Leu residues (**109**, Scheme 24).

**Scheme 25:**
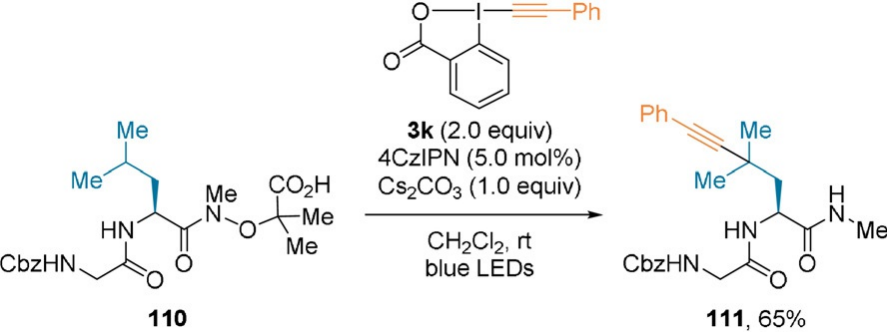
Photoinduced alkynylation by HAT.

Later, the Leonori group described a photoinduced remote H‐atom transfer (HAT) strategy for the functionalization of amines and amides, such as Leu‐containing dipeptide **110**, by using hypervalent iodine reagents as SOMOphiles (Scheme 25).^[48]^ An electrophilic amidyl radical is proposed to be generated from activated precursor **110**. After 1,5‐HAT, the tertiary radical can add on the EBX reagent **3 k**, thereby generating the alkynylated dipeptide **111** in moderate yield.

### 4.2 Alanine

**Scheme 26:**
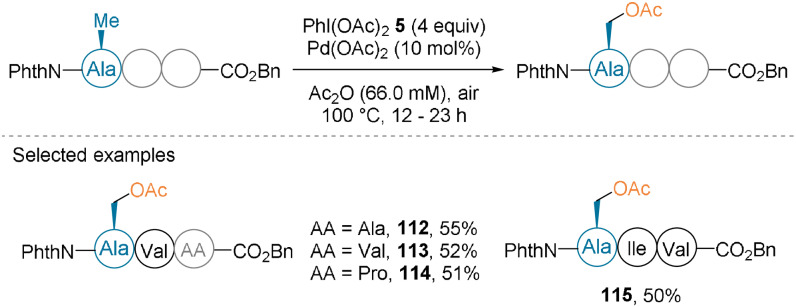
C(sp^3^)−H acetoxylation of tripeptides.

The Yu group described the acetoxylation of Ala‐containing tripeptides with a phthalimide group at the N‐terminus using PIDA (**5**) as an oxidant.^[49]^ The side chains of N‐terminal Ala residues were oxidized using 10 mol % Pd(OAc)_2_ in acetic anhydride at 100 °C (Scheme 26). Although moderate yields were obtained for products **112**–**115**, selectivity for the N‐terminal Ala residue, even in the presence of a C‐terminal Ala residue, was observed (**112**).

## 5 Peptide Termini

Direct and selective functionalization of peptide termini is an attractive method to achieve biomolecule modification.[^20^, ^50^] It allows a single site‐selective modification of native peptides without the need to introduce or change an amino acid residue.

### 5.1 C‐terminus

Photoredox‐catalyzed decarboxylative transformations are especially attractive for the functionalization of C‐termini.^[3e]^ They allow the rapid generation of reactive species under mild reaction conditions with high selectivity for the C‐terminus, as this site is easier to decarboxylate under oxidative conditions compared to side chains.

**Scheme 27:**
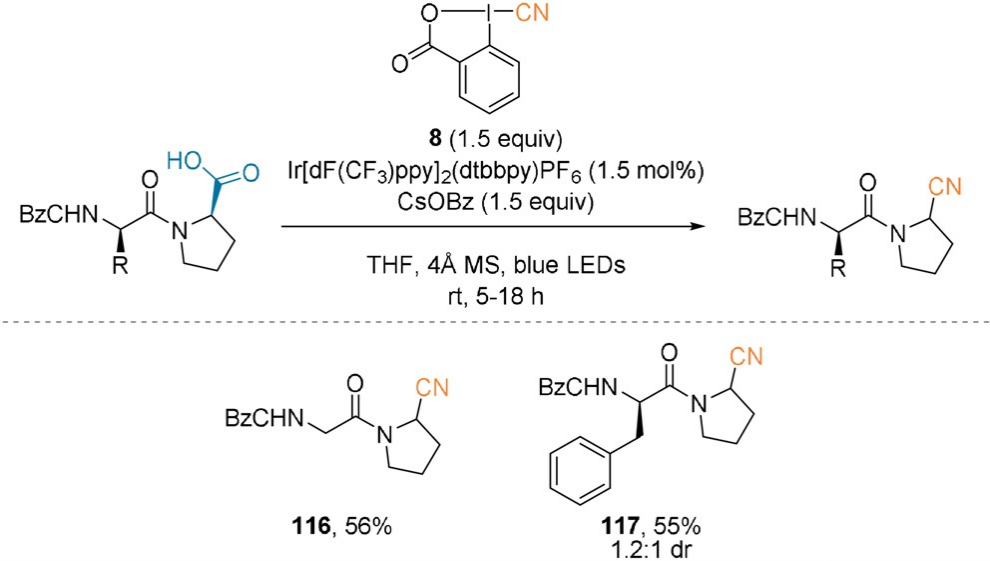
Decarboxylative cyanation using CBX (**8**).

EBX reagents have been used for the decarboxylative photoredox‐catalyzed alkynylation of amino acids.^[51]^ Our group developed an iridium‐catalyzed method^[51b]^ that was later extended to a decarboxylative cyanation of amino acids and peptides.^[52]^ Cyanobenziodoxolone (CBX, **8**), developed by Zhdankin et al.,^[53]^ was used along with catalytic amounts of Ir[dF(CF_3_)ppy]_2_(dtbbpy)PF_6_ in the presence of cesium benzoate and molecular sieves (Scheme 27). The reaction was applied to dipeptides to give cyano amides such as **116** and **117**.

**Scheme 28:**
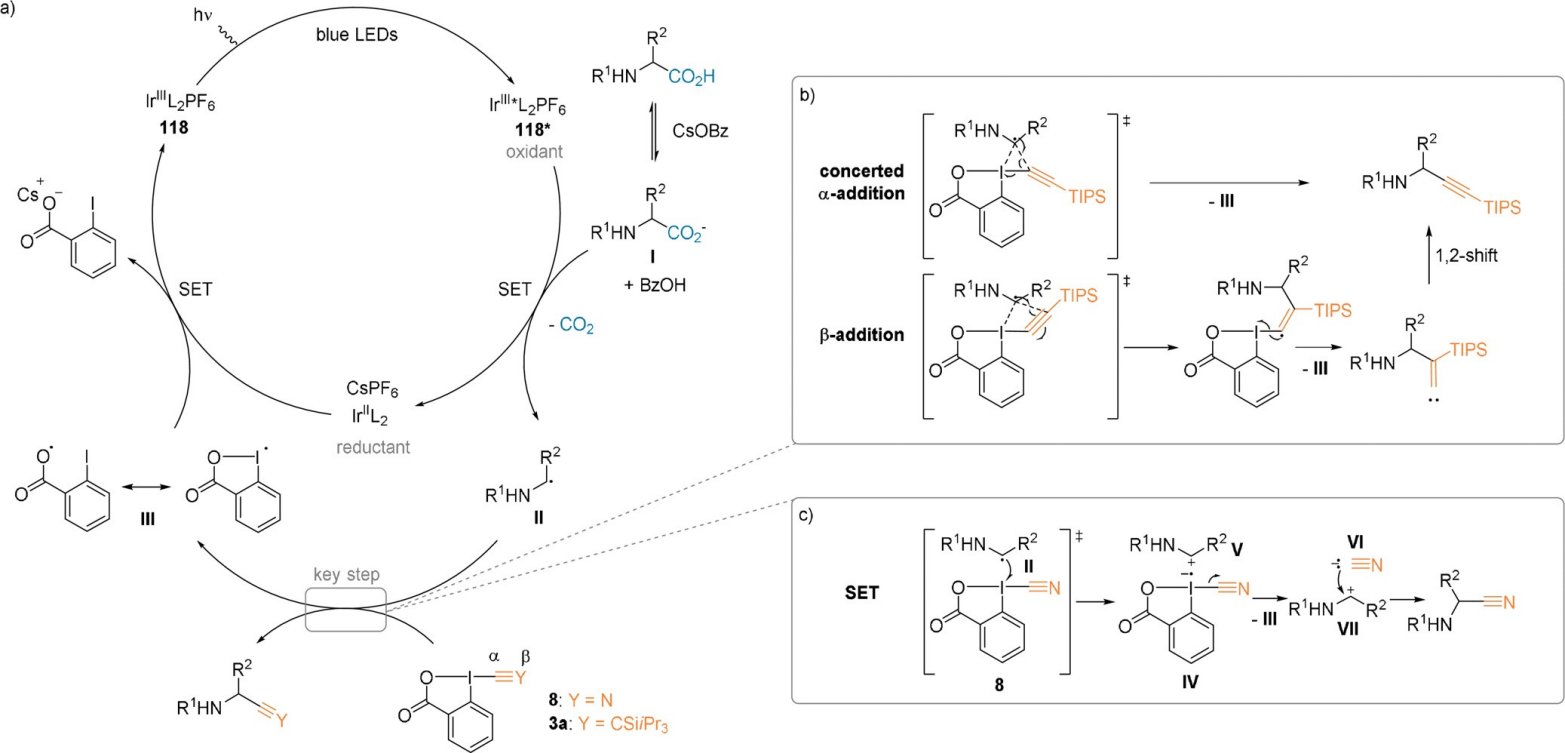
a) General catalytic cycle. Proposed mechanisms for b) alkynylation and c) cyanation.

Experimental and computational studies reveal that the alkynylation and cyanation reactions are not going through the same intermediates (Scheme 28).[^51b^, ^52^] The common catalytic cycle starts with the excitation of **118** (Scheme 28 a). A single‐electron transfer (SET) between 118* and the in situ generated carboxylate **I** occurs, thereby generating the nucleophilic radical **II** after decarboxylation. The alkynylation then undergoes a full radical pathway via the α‐ or β‐addition of **II** to the reagent **3 a** (Scheme 28 b). In contrast, another SETfrom radical **II** to CBX **8** is believed to yield the radical anion **IV** and iminium **V** (Scheme 28 c). Collapse of the radical anion to form cyanide **VI**, followed by recombination with the carbocation **VII**, then leads to the cyanated product.

**Scheme 29:**
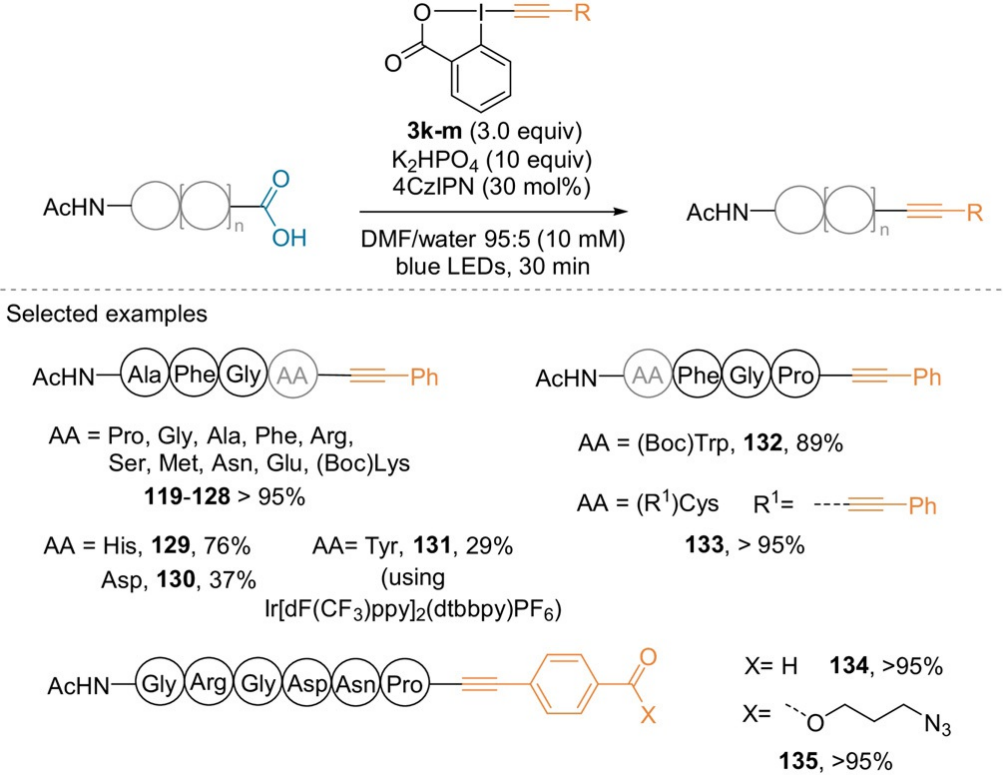
Decarboxylative alkynylation using R‐EBX reagents **3 k**–**m**.

In 2019, our group extended the scope of decarboxylative alkynylation to peptides.^[54]^ The reaction proceeded under mild, metal‐free conditions in 30 minutes at room temperature in a DMF/water mixture. In general, a wide range of C‐ and N‐terminal amino acids were compatible with the reaction (**119**–**133**, Scheme 29). Reactivity in other cases can be improved using either side‐chain protections (**128**, **132**) or a different catalyst (**131**). In the presence of Cys, both the thiol group and the C‐terminus were alkynylated (**133**). Functionalization of bioactive hexapeptide quantitatively yielded products containing reactive handles, such as an aldehyde or an azide (**134**, **135**).

**Scheme 30:**
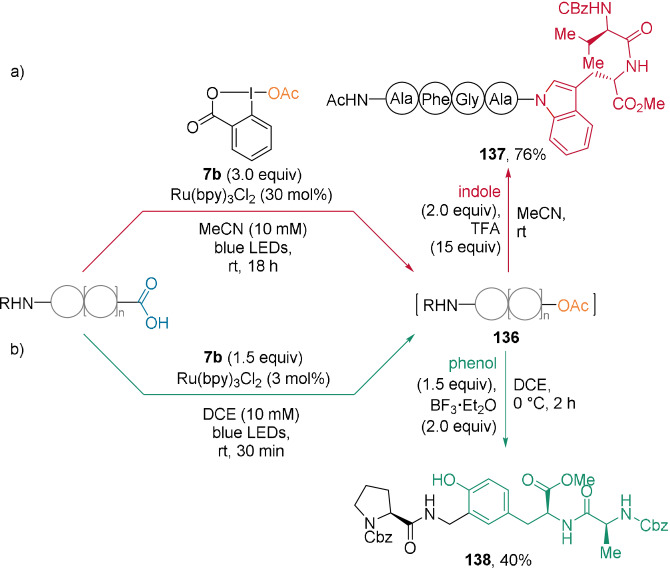
Selected examples of decarboxylative introduction of proteinogenic a) indoles and b) phenols on the C‐terminus of various peptides.

In 2021, our group introduced a photoredox‐catalyzed oxidative decarboxylative reaction using acetoxybenziodoxole (BI‐OAc, **7 b**; Scheme 30).^[55]^ This allowed the transfer of the acetoxy group to peptides, thereby forming intermediates **136**. The generated N,O‐acetals could not be isolated, but were trapped with phenol or indole nucleophiles. For example, dipeptides containing a proteinogenic phenol or indole were introduced, which provided unprecedented unnatural peptides **137** and **138**. Alternatively, in the presence of an alcohol, for example, serine or threonine residues, structurally diverse and stable N,O‐acetals were formed.

**Scheme 31:**
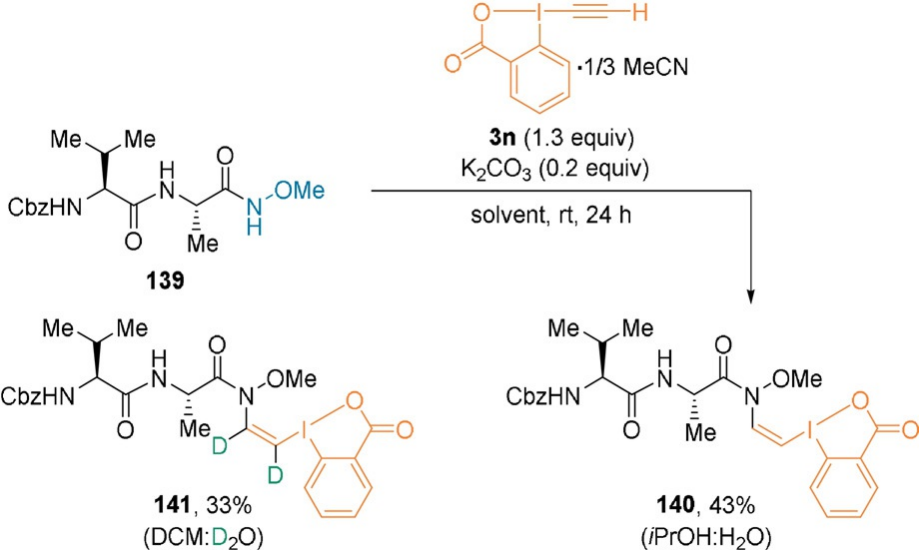
Synthesis of *cis*‐β‐*N*‐MeO‐amide‐VBX dipeptides.

In 2019, Tada, Itoh, and co‐workers introduced the H‐EBX reagent **3 n**, stabilized by MeCN.^[56]^ **3 n** was used to functionalize *O*‐methylhydroxamic acids derived from carboxylic acids (Scheme 31).^[57]^ For example, C‐terminal‐modified dipeptide **139** was used to yield *cis*‐β‐*N*‐MeO‐amide‐VBX **140** in the presence of water and a catalytic amount of base. In the presence of deuterium oxide, deuterium incorporation occurred at the vinyl positions, yielding **141**.

### 5.2 N‐terminus

**Scheme 32:**
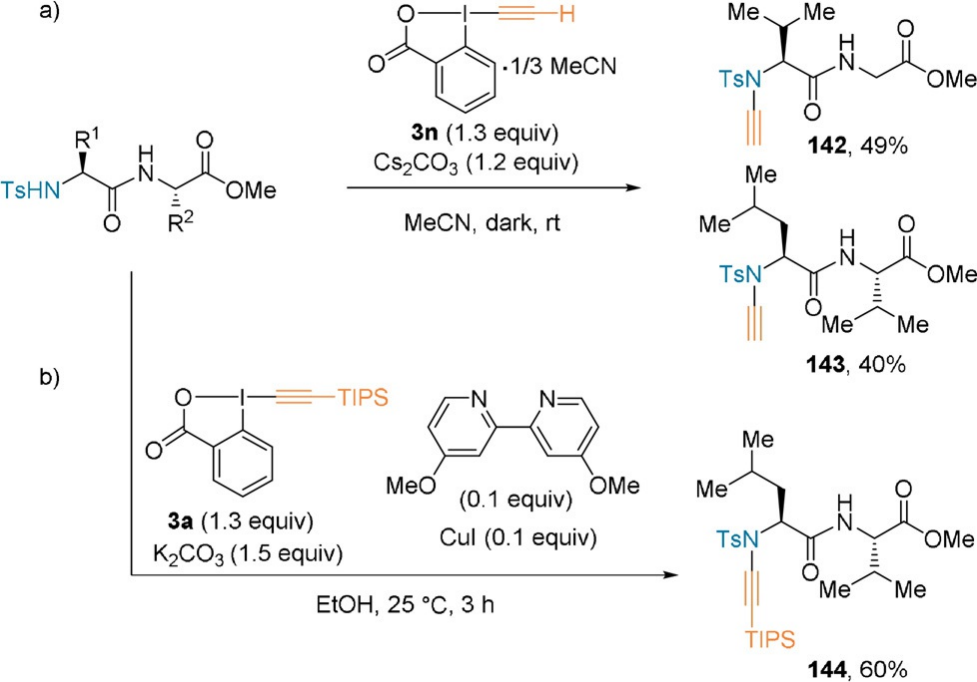
N‐terminus alkynylation of dipeptides using **3 n** and **3 a**.

Besides C‐terminal modification, **3 n** also reacted with a tosyl‐protected N‐terminus to yield alkynylated dipeptides **142** and **143** when an excess of base was used (Scheme 32 a).^[56]^ Although the scope was first limited to unsubstituted EBX, this drawback was addressed by the use of a copper catalyst.^[58]^ Reagents bearing various substituents were added at the N‐terminus of amino acids, while TIPS‐EBX **3 a** was used to obtain functionalized dipeptide **144** (Scheme 32 b).

## 6 Summary and Outlook

This Minireview summarizes applications of hypervalent iodine reagents for the modification of peptides and proteins. They have been used as oxidants or electrophilic transfer reagents. A wide range of reagents have been designed and applied to a variety of amino acid side chains for bioconjugation, peptide stapling, or proteome‐wide Cys profiling. Many methods achieve high site‐selectivity and are performed under biocompatible conditions. This has allowed applications on complex substrates, such as antibodies, proteins, and in living cells.

Although great advances have been achieved in the field, challenges still remain. For example, many methods are limited to amino acids or short peptides and can be only used in organic solvents. In addition, these biomolecule modifications are currently limited to oxidation, fluoroalkylation, thiolation, alkynylation, cyanation, azidation, and alkoxylation. The development of new reagents is needed to introduce further diversity. This requires new synthetic methods to give easy access to novel hypervalent iodine compounds that combine aqueous solubility, reactivity, and stability.

Nevertheless, these discoveries are still very recent. From the advantages offered by hypervalent iodine reagents, improvements can be expected in the near future, leading to an increased use of these reactants for applications in biochemistry.

## Conflict of interest

The authors declare no conflict of interest.

## Biographical Information

*Emmanuelle M. D. Allouche received a chemical engineering degree in 2014 from ENSICAEN conjointly with an MSc from the Université Caen‐Normandie (France). She completed her PhD in 2019 with Prof. André B. Charette (Université de Montréal, Canada). Her research focused on the synthesis of polysubstituted cyclopropanes by Suzuki coupling or using donor diazo compounds produced in batch or continuous flow. She joined the group of Prof. Jerome Waser in 2020 as a postdoctoral fellow and currently works on the azidation of biomolecules*.

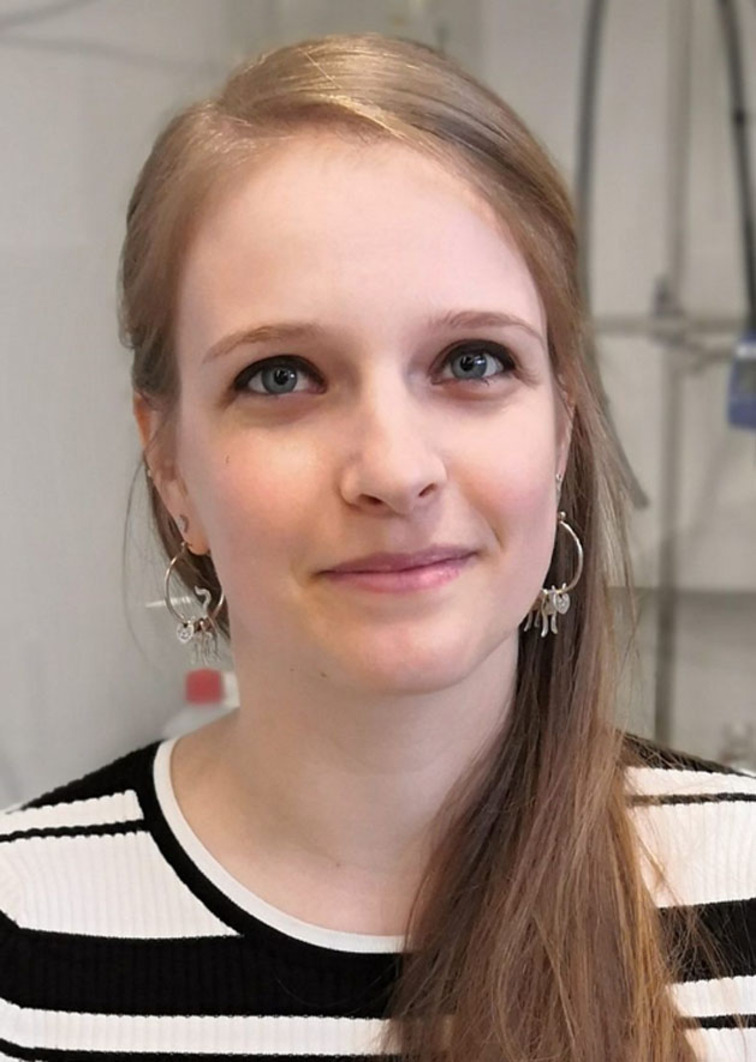

## Biographical Information

*Elija Grinhagena obtained her MSci from the University of Glasgow in 2018. During her degree she undertook an industrial placement year at F. Hoffmann‐La Roche (Basel, Switzerland). She then returned to complete her final year project with Prof. Stephen Clark in the field of peptide mimic synthesis. In 2019, she joined the Prof. Jerome Waser group, where she is currently completing her doctoral studies and working on the development of peptide stapling methods using hypervalent iodine reagents*.

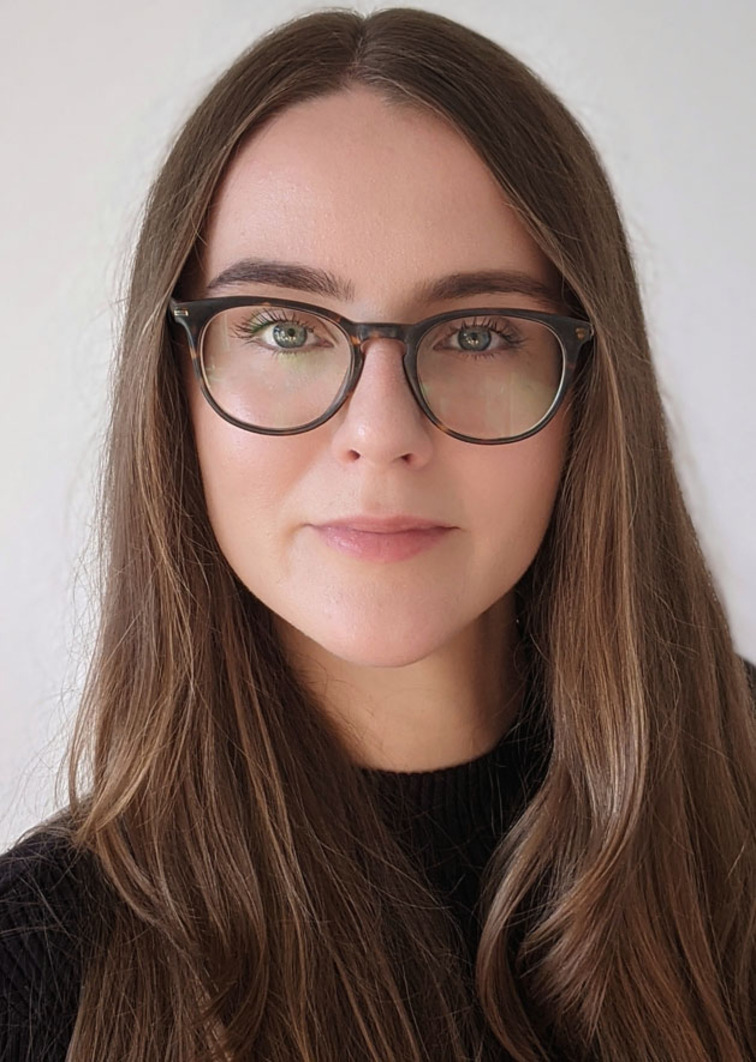

## Biographical Information

*Jerome Waser was born in Sierre, Valais, Switzerland. He studied chemistry at ETH Zurich, where he obtained his PhD in 2006 with Prof. Erick M. Carreira. In 2006, he joined Prof. Barry M. Trost at Stanford University as a SNF postdoctoral fellow. Since October 2007 he has been professor of organic chemistry at the Ecole Polytechnique Fédérale de Lausanne (EPFL), where he was promoted to full professor in 2019. Since 2020, he has also been co‐director of the NCCR Catalysis of the Swiss National Science Foundation*.